# Photo‐RAFT Polymerization Under Microwatt Irradiation via Unimolecular Photoinduced Electron Transfer

**DOI:** 10.1002/anie.202424225

**Published:** 2025-03-17

**Authors:** Giovanni Lissandrini, Davide Zeppilli, Francesca Lorandi, Krzysztof Matyjaszewski, Abdirisak A. Isse, Laura Orian, Marco Fantin

**Affiliations:** ^1^ Department of Chemical Sciences University of Padova Via Marzolo 1 Padova 35131 Italy; ^2^ Department of Chemistry Carnegie Mellon University 4400 Fifth Avenue Pittsburgh Pennsylvania 15213 USA; ^3^ Present address: Laboratory for Macromolecular and Organic Chemistry Department of Chemical Sciences University of Padova Via Marzolo 1 Padova 35131 Italy

**Keywords:** AND logic, Base, Deprotonation, PET‐RAFT, Weak light

## Abstract

Photoinduced radical addition fragmentation chain transfer (PET‐RAFT) polymerization typically requires high light intensity (>5 mW cm^−^^2^), limiting energy efficiency, and scalability. We demonstrate that adding a base to PET‐RAFT systems drastically enhances the reactivity of acidic chain transfer agents (CTAs) with Zn‐based photocatalysts (Zn porphyrin and Zn phthalocyanine). This approach enables complete polymerization under microwatt light intensity (0.25 mW cm^−^^2^), a significant improvement over traditional PET‐RAFT, which showed no conversion under the same conditions. Both acrylates and methacrylates polymerized efficiently with excellent chain‐end fidelity. Reactivity was triggered chemically (via base addition) or electrochemically (via electrolytic reduction). Mechanistic studies reveal that base addition promotes a CTA‐Zn photocatalyst complex, shifting the activation from bimolecular to more efficient unimolecular PET‐RAFT.

## Graphical abstract

Adding a base to PET‐RAFT polymerization significantly enhances the reactivity of acidic CTAs with Zn‐based photocatalysts, enabling polymerization under low light intensity (0.25 mW cm^−^^2^). This approach outperforms traditional PET‐RAFT, which showed no conversion under the same conditions. Both acrylates and methacrylates polymerized efficiently. Mechanistic studies suggest base addition shifts activation from bimolecular to more efficient unimolecular PET‐RAFT.

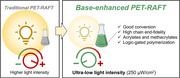

## Introduction

Reversible deactivation radical polymerizations (RDRPs) have transformed the synthesis of macromolecules, providing powerful tools for controlling architecture, composition, and chain length distribution.^[^ ^1^, ^2^, ^3^ ^]^ RDRP establishes a dynamic equilibrium between propagating radicals and dormant species, ensuring that growing radicals are deactivated before undergoing termination reactions and react with only a limited number of monomeric units. This allows polymerization to be reinitiated at any time by introducing additional monomers and initiators, enabling the production of block copolymers or complex polymeric architectures.^[^ ^4^ ^]^

ATRP (atom transfer radical polymerization) and RAFT (reversible addition‐fragmentation chain transfer) are two of the most versatile and successful RDRP methods.^[^ ^5^ ^]^ RAFT uses a chain transfer agent (CTA), typically a dithiobenzoate or trithiocarbonate, to regulate polymer chain transfer.^[^ ^6^ ^]^ CTAs with a carboxylic acid (COOH) group have achieved the highest commercial success due to their easy synthesis, odor control, enhanced solubility, and effective polymerization control.^[^ ^7^ ^]^

Traditional RAFT polymerization is triggered by thermal initiators (e.g., peroxides or diazo initiators).^[^ ^8^ ^]^ However, over the years various external stimuli have been successfully applied to RAFT polymerization, including photochemical,^[^ ^9^ ^]^ electrochemical,^[^ ^10^, ^11^ ^]^ and chemical methods.^[^ ^8^ ^]^ All these approaches enable safe mild reaction conditions and excellent reaction control.

Photoinduced RAFT can proceed through two mechanisms: i) photoinduced iniferter polymerization, where light‐driven photolysis of a CTA occurs in the absence of a photoinitiator, typically under UV or blue light,^[^ ^12^, ^13^ ^]^ and ii) photoinduced electron/energy transfer PET‐RAFT polymerization, which relies on electron/energy transfer from a photocatalyst (PC) absorbing visible light to a CTA.^[^ ^14^ ^]^ PET‐RAFT has been shown to proceed via an electron transfer pathway with common PCs like zinc tetraphenylporphyrin (ZnTPP).^[^ ^15^ ^]^ PET‐RAFT is of interest due to its ease of use and functional group tolerance.

**Scheme 1:**
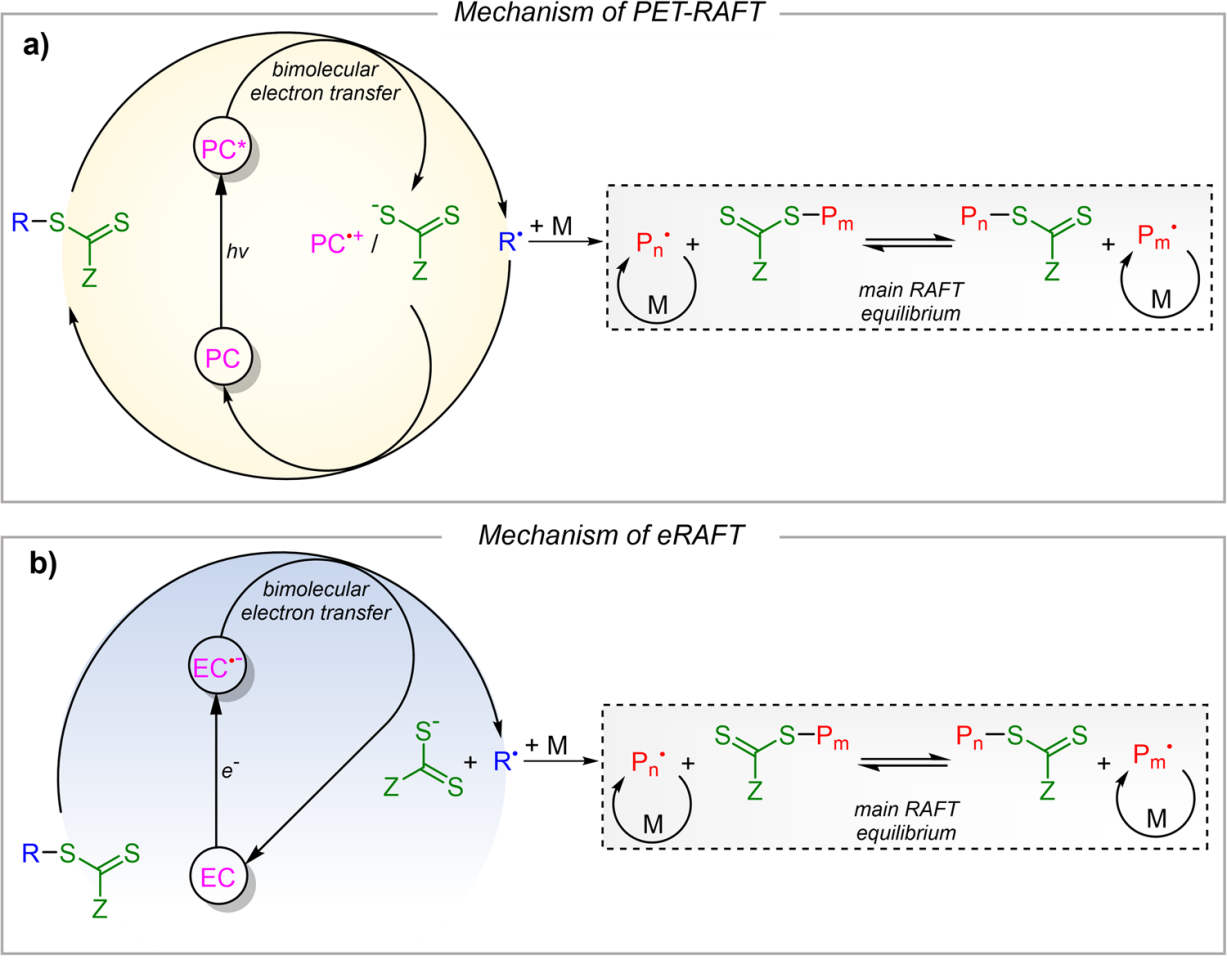
Mechanism of traditional a) PET‐RAFT and b) eRAFT polymerization via bimolecular electron transfer.

The mechanism of PET‐RAFT polymerization via electron transfer is illustrated in Scheme 1a. A photosensitizer absorbs light, and the resulting excited‐state transfers an electron to the CTA via bimolecular electron transfer. This leads to a reductive bond cleavage in the CTA, generating a radical (R^•^), which could be a small molecule or a polymer radical if the CTA is capped on a polymer chain. The resulting radical initiates polymerization by reacting with the monomer (M) and continues to exchange with the remaining CTA, following the primary RAFT equilibrium.

Photoinduced RAFT typically requires substantial light irradiation to initiate polymerization, with monochromatic light intensities of 5 mW cm^−^^2^ or more,^[^ ^16^, ^17^, ^18^ ^]^ and rarely using intensities around or below 1 mW cm^−^^2^.^[^ ^19^ ^]^ From a recent review,^[^ ^9^ ^]^ an average of 18.4 mW cm^−^^2^ for PET‐RAFT reactions (arithmetic average) was calculated, with a geometric average of 4.3 mW cm^−^^2^. These values, while not extremely high, represent energy inefficiency, which can hinder reaction scale‐up, particularly with large volumes, due to heat dissipation or poor light penetration. High irradiation intensities suggest that only a small fraction of photons contribute to generate propagating radicals, partly due to the limited lifetime or slow electron transfer of excited‐state photocatalysts, limiting the efficiency of electron transfer from PC* to the CTA.

Another RAFT activation method involves acids. As reported by Anastasaki, Coote et al.,^[^ ^20^ ^]^ strong or weak acids (e.g., sulfuric or citric acid) enable well‐controlled polymerization of many monomers in aqueous and non‐aqueous media, yielding polymers with dispersity *Ð* < 1.2 and high terminal group fidelity.^[^ ^21^ ^]^ Despite adding up to 10 equivalents of acid relative to the CTA, the system remains stable without CTA decomposition.^[^ ^22^ ^]^

Electrochemical methods offer an environmentally friendly alternative for RAFT polymerization, eliminating the need for chemical reducing agents or thermal initiators and enabling real‐time monitoring. However, their application is constrained by the complex electrochemistry of RAFT agents, which undergo irreversible side reactions during direct electrolysis.^[^ ^23^, ^24^ ^]^ Using mediators, such as electrocatalysts (ECs), allows indirect reduction of the CTA by electrogenerated radical anions (Scheme 1b).^[^ ^11^ ^]^ This method enables efficient polymerization, as each electron injected directly forms a radical that drives the polymerization.

This study revealed a surprising reactivity enhancement in PET‐RAFT polymerization when a base was added to catalytic systems composed of acidic CTAs and Zn‐based photocatalysts. Under ambient light (∼0.25 mW cm^−^^2^), high conversion occurred within hours, whereas traditional (neutral) conditions resulted in no polymerization. Polymerization required the simultaneous presence of both a base and light, with a synergistic effect that we demonstrated to follow an “AND” logic.^[^ ^25^ ^]^

PET‐RAFT polymerization could be promoted through chemical (base addition) or electrochemical (electrolytic catalyst reduction) methods, enabling precise external control with a switchable catalyst, which was previously observed for magnetic switching.^[^ ^26^ ^]^

This base‐enhanced approach enables both acrylate and methacrylate polymerization while preserving chain‐end fidelity for block copolymers. Mechanistic studies suggest that Zn‐based photocatalysts interact with RAFT agent carboxyl groups, forming a CTA–PC precomplex that shifts the mechanism from bimolecular to more efficient unimolecular electron transfer. Previous studies reported pH‐induced aggregation in water‐soluble ZnTPP derivatives.^[^ ^25^ ^]^ The key finding is a highly efficient, safe photo‐RAFT polymerization method using microwatt power irradiation, with reactivity triggered by base addition.

## Results and Discussion

### PET‐RAFT With Protonated and Deprotonated CTAs

**Figure 1:**
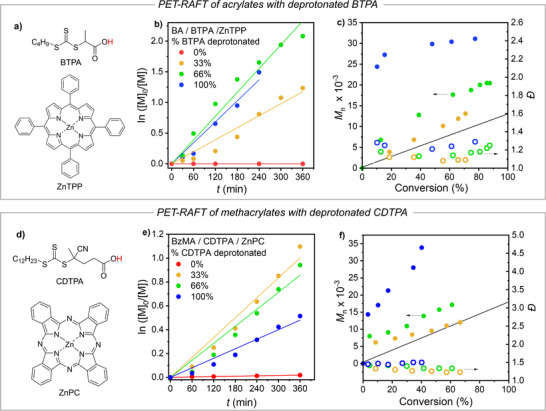
PET‐RAFT polymerizations of 50% (v/v) monomer in DMSO at 50 °C under 0.25 mW cm^−2^ irradiation at different degrees of CTA deprotonation (labeled in the Figures). a–c) PET‐RAFT of BA with the ZnTPP/BTPA catalytic system: a) Structures of BTPA and ZnTPP, b) kinetic plots, and c) MW and *Đ* trends. d–f) PET‐RAFT of BzMA with the ZnPC/CDTPA catalytic system: d) Structures of CDTPA and ZnPC, e) kinetic plots, and f) MW and *Đ* trends. Complete reaction conditions are described in Table 1. The straight lines in c) and f) stand for the theoretical molecular weights.

We investigated base‐enhanced PET‐RAFT polymerization by examining the effect of a base on butyl acrylate (BA) polymerization in the presence of a common photocatalyst‐CTA pair composed of zinc tetraphenylporphyrin (ZnTPP) and 2‐(butylthiocarbonothioylthio)propanoic acid (BTPA) (see structures in Figure 1a).^[^ ^27^ ^]^ Under weak white LED light (0.25 mW cm^−^^2^) and shielding to block ambient light (Figures S3–S4), no polymerization occurred with fully protonated BTPA, suggesting that traditional bimolecular PET‐RAFT does not proceed at such low light intensity (Table 1, Entry 1 and Figure 1b). However, upon progressive deprotonation of the CTA with 0.33, 0.66, and 1 equiv. of *n‐*Bu_4_NOH, progressively faster polymerization was observed (polymerization rate was similar with 0.66, and 1 equiv. *n‐*Bu_4_NOH, corresponding to 66% and 100% deprotonated BTPA).

**Table 1:** PET‐RAFT polymerizations of BA and BzMA with CTA at different degrees of deprotonation under 0.25 mW cm^−2^ white light.

Entry^a)^	M/CTA	% CTA deprotonated^b)^	% Conv^c)^	*k*_p,app_ (h^−1^)^d)^	*M*_n,GPC_ ^e)^	*M*_n,th_ ^f)^	*I*_eff_ ^g)^	*Đ* ^e)^
1	BA/BTPA^h)^	0	<5	0	–	–	–	–
2	–	33	71	0.20	13 100	9300	0.71	1.09
3	–	66	88	0.39	20 500	11 500	0.56	1.25
4^i)^	–	100	78	0.34	31 100	10 200	0.26	1.29
5	BzMA/CDTPA^j)^	0	<5	0	–	–	–	–
6	–	33	67	0.17	12 000	12 100	1.01	1.23
7	–	66	61	0.14	17 200	11 200	0.65	1.34
8	–	100	40	0.08	33 900	7500	0.22	1.50

^a)^ General polymerization conditions: *T* = 50 °C, 50% (v/v) M in DMSO, *V*_tot_ = 10 mL;

^b)^ Via addition of *n‐*Bu_4_NOH;

^c)^ Reaction time = 6 h;

^d)^ The slope of the ln([M]_0_/[M]) versus time plot;

^e)^ Measured via GPC with DMF eluent at 60 °;C

^f)^ *M*_n,th_ = *M*_w,CTA_ + ([M]/[CTA])*M*_w,M_ × % conv;

^g)^ *I*_eff_ = *M*_n,th_/*M*_n,GPC_;

^h)^ [BA]:[BTPA]:[ZnTPP] = 100:1:0.02;

^i)^ 4 h polymerization time;

^j)^ [BzMA]:[CDTPA]:[ZnPC] = 100:1:0.02.

Deprotonation of BTPA significantly affected both polymerization rate and control. At 33% CTA deprotonation, excellent control (*Đ* < 1.1) and minimal deviation between experimental and theoretical molecular weights (MW) were observed (Figure 1c). At 100% deprotonation, dispersity increased to ∼1.3, and molecular weight agreement worsened, indicating that complete deprotonation hindered chain transfer (see further details on chain transfer rates in the last section of this manuscript). Optimal conditions were found at 33% deprotonation, achieving both fast polymerization and excellent control.

Control experiments confirmed that light, CTA, base, and photocatalysts were all necessary to trigger PET‐RAFT polymerization (Table S1). This also ruled out the possibility of a photoiniferter mechanism.^[^ ^28^ ^]^ Both base and light were required to activate the PC/CTA system, making the polymerization mechanism controlled by an “AND” logic: both inputs—light and base—must be present to synergistically trigger polymerization under weak light irradiation (see more details below).

A similar rate increase upon CTA deprotonation was observed for the PET‐RAFT of benzyl methacrylate (BzMA) with the CDTPA/ZnPC catalytic system (see structures in Figure 1d). Again, under 0.25 mW cm^−2^ irradiation, no polymerization was observed with fully protonated CDTPA (Figure 1e), whereas relatively fast polymerization was observed via PET in the presence of 33% deprotonated CDTPA (Table 1, Entries 5 and 6, and Figure 1e–f). Polymerization reached ca. 70% conversion in 6 h, with good control of molecular weight and low dispersity.

Deprotonating CDTPA beyond 33% did not enhance polymerization, but instead slowed the process and reduced control, likely due to slow CTA fragmentation (see below). This confirms that ∼33% CTA deprotonation is optimal. Further 2X rate acceleration could be achieved by pairing CDTPA with the more reducing photocatalyst ZnTPP instead of ZnPC (Figure S11).

Other methacrylate monomers with polar and potentially complexing groups such as 2‐(dimethylamino)ethyl methacrylate (DMAEMA) and (hydroxyethyl)methacrylate (HEMA) were polymerized at 33% CDTPA deprotonation with good control over dispersity and MWs (Figure S12). In summary, partial CTA deprotonation (33%) in the presence of Zn‐based PCs effectively activated PET‐RAFT polymerization under weak light, achieving a good rate and excellent control.

### Externally Gated Polymerization by a Combined Photo‐Electrochemical Approach

Since RAFT agents were activated by a simple acid/base reaction, we aimed to develop a system for fully external control of polymerization. This approach uses external stimuli, instead of a chemical base, to trigger PET‐RAFT polymerization with acidic CTAs.^[^ ^29^ ^]^

**Figure 2:**
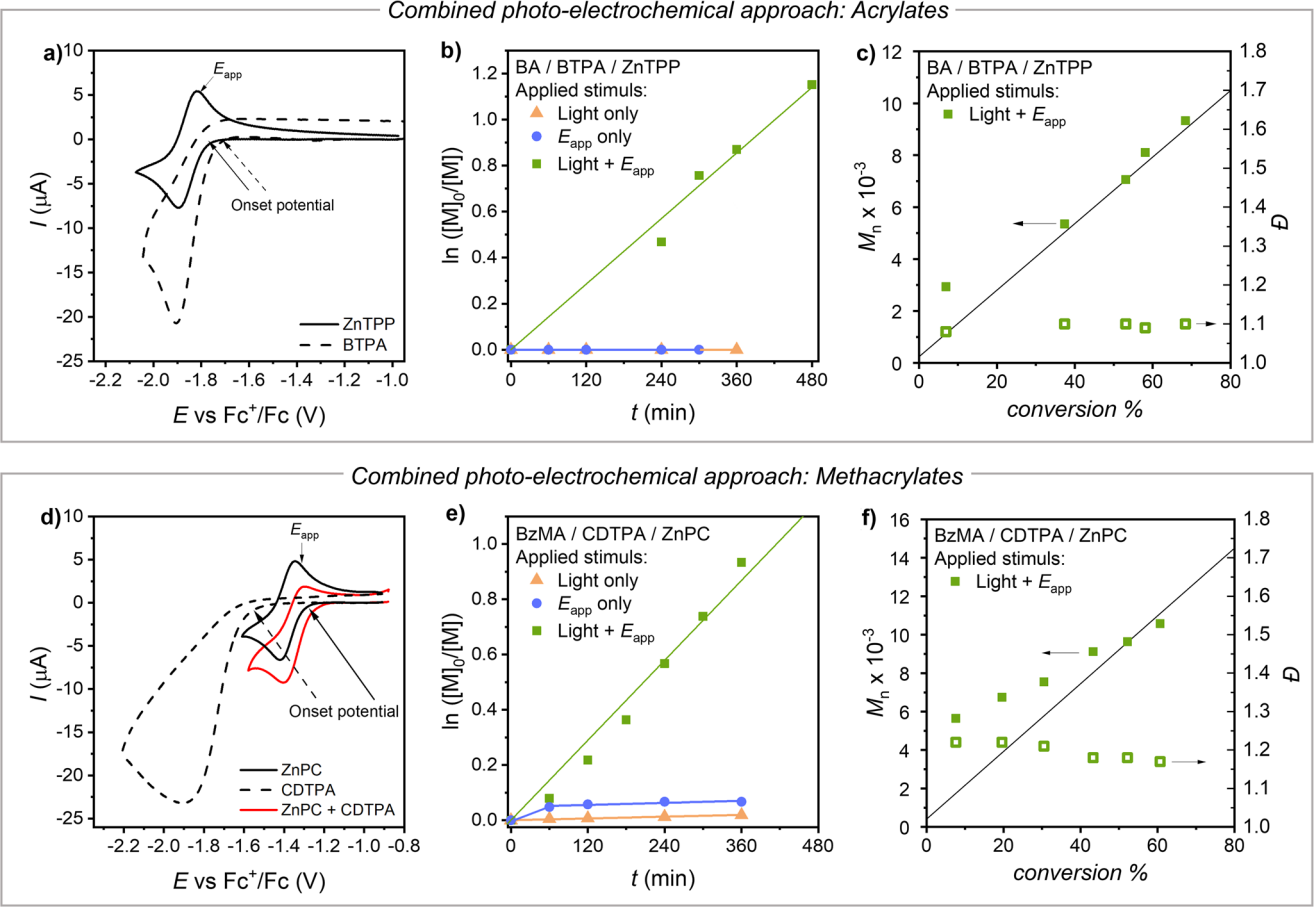
CV of a) 2 × 10^−3^ M BTPA and 5 × 10^−4^ M ZnTPP and d) 2 × 10^−3^ M CDTPA, 5 × 10^−4^ M ZnPC, and 5 × 10^−4^ M ZnPC + 2 × 10^−3^ M CDTPA, in DMSO + 0.1 M *n*‐Bu_4_NBF_4_ recorded on a Pt disk electrode (*d* = 3 mm) at 0.1 V s^−1^ and *T* = 25 °C. Kinetic plots and trends of MW and dispersity of RAFT polymerizations of b) and c) BA 50% (v/v) in DMSO using the catalytic system ZnTPP/BTPA and e) and f) BzMA at 50% (v/v) in DMSO using the catalytic system ZnPC/CDTPA: comparison between electrochemical, photochemical, and photo‐electrochemical methods (see polymerization details Table 2). The solid lines in c) and f) indicate the theoretical Mn.

We tested whether an electrochemical method could activate the CTAs through deprotonation under reducing conditions, generating anions and deprotonating the CTAs (see also Figures S6–S7 and related discussion). First, we investigated the voltammetric properties of PCs and CTAs on a Pt disk electrode. Cyclic voltammetry (CV) of ZnTPP in DMSO showed a reversible peak couple, with a half‐wave potential *E*_1/2,ZnTPP_  = −1.86 V versus Fc^+^/Fc (Figure 2a). The observed peak couple is attributed to a reversible one‐electron transfer involving the ZnTPP/ZnTPP^●−^ redox couple. Reduction of BTPA occurred at potentials similar to *E*_1/2_ of ZnTPP, but with an irreversible peak (*E*_p_ = ‐1.91 V vs Fc^+^/Fc at scan rate *v* = 0.1 V s^−1^), indicating that BTPA decomposes upon electrochemical reduction as was previously reported for several CTAs.^[^ ^24^ ^]^ Further electrochemical characterizations of BTPA are reported in Figure S7.

After confirming that light alone (0.25 mW cm^−2^, no *E*_app_) did not trigger polymerization with fully protonated BTPA (orange triangles in Figure 2b, and Entry 2, Table 2), both stimuli (*E*_app_ and 0.25 mW cm^−2^ light) were applied to the system. This successfully triggered a well‐controlled PET‐RAFT (Figure 2b, and Entry 3, Table 2). The electrochemical stimulus was responsible for the deprotonation of the CTA, allowing it to interact with the ZnTPP catalyst and be rapidly activated by the weak light irradiation. This dual‐controlled polymerization scheme corresponds to a fully externally gated polymerization, operating on an “AND” logic, where both external stimuli—light and *E*_app_—must be simultaneously applied to trigger polymerization. This could be exploited for selective temporal polymerization control (see below).

**Table 2:** Comparison between electrochemical, photochemical, and photo–electrochemical methods for the PET‐RAFT of BA and BzMA in DMSO.

Entry^a)^	M/CTA/Catalyst	Applied Stimuli	*k*_p app_ (min^−1^)^b)^	Conv (%)^c)^	*M*_n_ ^d)^	*M*_n,th_	*I*_eff_ ^e)^	*Đ* ^d)^
1^f)^	BA/BTPA/ZnTPP	*E*_app_	–	<5	–	–	–	–
2^f)^	–	Light	–	<5	–	–	–	–
3^f)^	–	Light + *E*_app_	0.15	59	8100	7700	0.95	1.09
4^g)^	BzMA/CDTPA/ZnPC	*E*_app_	–	<5	–	–	–	–
5^g)^	–	Light	–	<5	–	–	–	–
6^g)^	–	Light + *E*_app_	0.19	61	10 600	11 000	1.04	1.17

^a)^ General polymerization conditions: *T* = 50 °C, 50% (v/v) M in DMSO + 0.1 M *n*‐Bu_4_NBF_4_, *V*_tot_ = 10 mL, *E*_app_ = *E*_1/2,Cat_ + 0.06 V, Light = 0.25 mW cm^−2^ white light, reaction time 6 h;

^b)^ The slope of the ln([M]_0_/[M]) versus time plot;

^c)^ Measured via ^1^H‐NMR;

^d)^ Measured via GPC with DMF eluent at 60 °C;

^e)^ *I*_eff_ = *M*_n,th_/*M*_n,GPC;_

^f)^ [M]:[CTA]:[Cat] = 100:1:0.02 with [M] = 3.27 M;

^g)^ [M]:[CTA]:[Cat] = 100:1:0.02 with [M] = 2.95 M.

PET‐RAFT polymerizations with electrochemically deprotonated CTA (Table 2) closely matched those with chemically deprotonated CTA (0.33 equiv. *n*‐Bu_4_NOH, Table 1, Entry 2). Both methods enabled PET‐RAFT polymerization with good rate and excellent control. To confirm their similarity, a polymerization was conducted using only electrical potential for 4 h, followed by light irradiation, which triggered immediate, rapid polymerization (Figure S8).

A similar photo‐electrochemical “AND” logic could be applied to the BzMA polymerization system, utilizing the ZnPC catalyst and CDTPA chain transfer agent. First, the voltammetric properties of this system were investigated at a Pt disk electrode. The CV of ZnPC in DMSO showed a reversible peak couple assigned to the ZnPC/ZnPC^●−^ redox couple, with *E*_1/2,ZnPC_ = −1.35 V versus Fc^+^/Fc (Figure 2d). CDTPA showed an irreversible cathodic peak at significantly more negative potentials (*E*_p_ = −1.91 V vs Fc^+^/Fc). In this case, ZnPC could be selectively reduced at the electrode before reaching the onset potential for the reduction of the CTA. Voltammetric reduction of ZnPC in the presence of the CTA caused a modification of the CV pattern, with an increase in the cathodic current and a decrease in the anodic current. This pattern is typical of an electrocatalytic cycle, according to the mechanism shown in the left side of Scheme 1b. The neutral catalyst ZnPC was reduced to ZnPC^●−^ at the electrode but was subsequently regenerated by homogenous reaction with the CTA. The regenerated ZnPC was then reduced again at the electrode, contributing to the enhancement of the cathodic current observed in the red trace in Figure 2d.

A series of polymerizations was set up with the BzMA monomer and the CDTPA/ZnPC system in an electrochemical cell with a bulk Pt mesh electrode. The application of *E*_app_ = *E*_1/2,ZnPC_ + 0.06 V = −1.29 V versus Fc^+^/Fc caused the bulk reduction of ZnPC and the consequent electrocatalytic reduction of CDTPA. After application of the electrochemical stimulus in complete darkness for 6 h, little or no polymerization was observed in solution (blue circles in Figure 2d, and Entry 4 in Table 2), indicating that this electrocatalytic system could not efficiently generate propagating radicals, similarly to the ZnTPP/BTPA system. Application of light only (0.25 mW cm^−2^) to fully protonated CDTPA/ZnPC did not trigger polymerization (orange triangles in Figure 2e, and Entry 5 in Table 2). Only the simultaneous application of both light and *E*_app_ could efficiently trigger the polymerization (green squares in Figure 2e, and Entry 6 in Table 2). In this case, the electrochemical stimulus was responsible for the deprotonation of the CTA (see Figures S13–S15), which then could be activated by the weak light irradiation. Since 0.25 mW cm^−2^ is a low light intensity commonly found in laboratory settings, we conducted the same polymerization mediated by ZnPC‐CDTPA on the benchtop of the fume hood without additional lighting. Essentially the same kinetics were observed under the hood light as with 0.25 mW cm^−2^ LEDs, demonstrating the extremely low energy requirement of our base‐enhanced PET‐RAFT process, that could proceed with good rate under ambient lighting (Table S3 and Figure S9).

**Figure 3:**
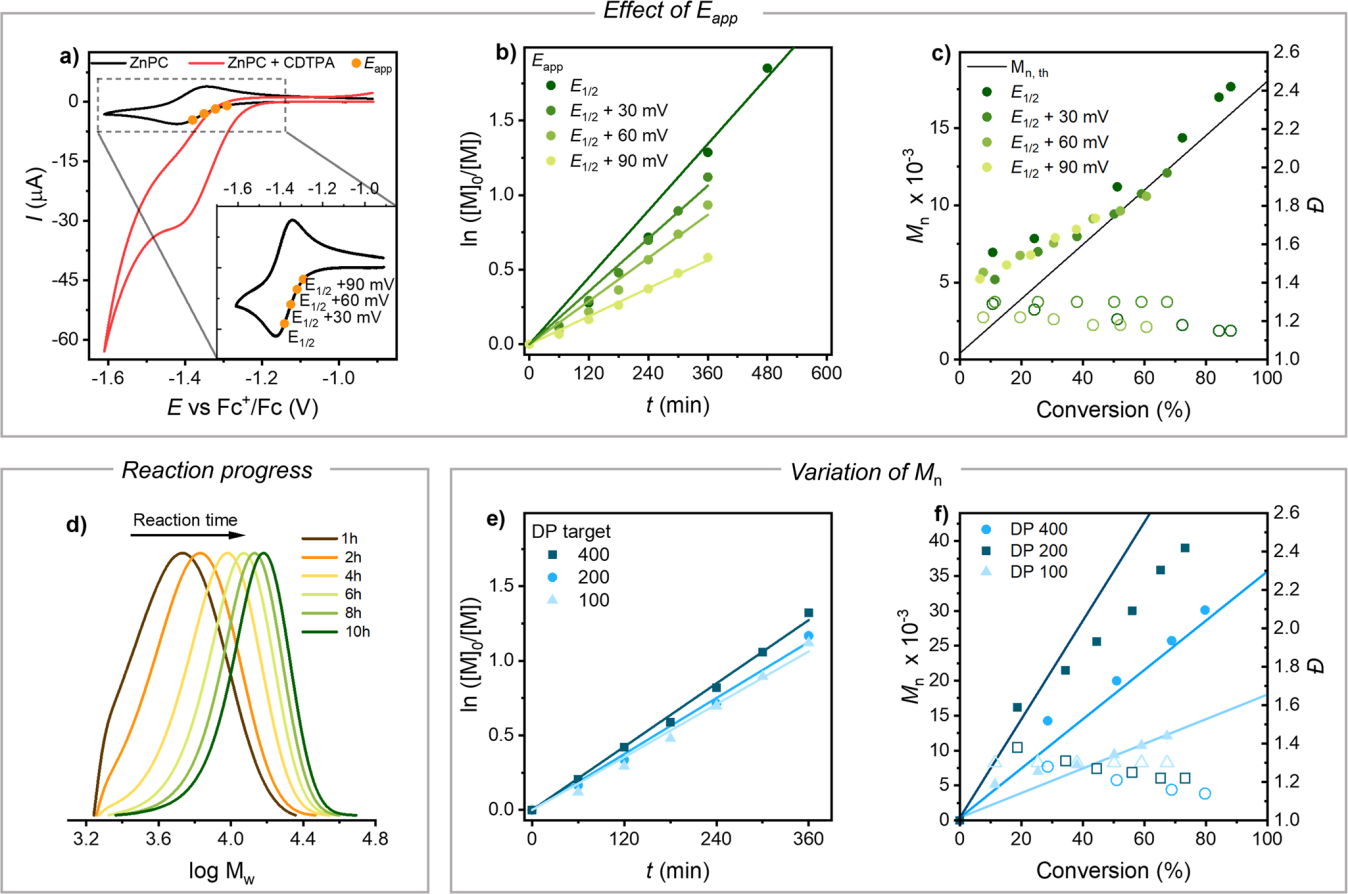
a) CV of 6 × 10^−4^ M ZnPC in the absence and in the presence of 2.9 × 10^−2^ M CDTPA in 50% (v/v) BzMA in DMSO + 0.1 M *n*‐Bu_4_NBF_4_, recorded on a Pt disk electrode at *v* = 0.1 V s^−1^. The inset highlights the *E*_app_ values selected for polymerizations (orange dots). b) Kinetic plots and c) variation of *M*_n_ (solid symbols) and dispersity (open symbols) for combined electrochemical/photochemical PET‐RAFT of BzMA at different values of *E*_app_. d) GPC traces representing the molar mass distribution during the polymerization of BzMA at *E*_app_ = *E*_1/2_ + 0.06 V. e) Kinetic plots and f) variation of *M*_n_ (solid symbols) and dispersity (open symbols) for combined electrochemical/photochemical PET‐RAFT of BzMA at different values of DP_target_, at *E*_app_ = *E*_1/2_ + 0.06 V. The straight lines in c) and f) stand for the theoretical molecular weights.

The effect of electrochemical parameters on polymerization rate was studied by varying *E*_app_ (between *E*_app_ = *E*_1/2,ZnPC_ +90 mV and *E*_app_ = *E*_1/2,ZnPC_, Figure 3a) under constant light irradiation of 0.25 mW cm^−2^. In the BzMA/CDTPA/ZnPC system, the polymerization rate increased with the application of more negative *E*_app_ (Figure 3b and Entries 1–4 in Table 3), consistent with faster electroreduction and faster formation of deprotonated CTA. This was also confirmed by the increasing electric charge *Q* passed with the application of more negative *E*_app_ (Table 3), as measured by chronoamperometry recorded during the polymerization (see example in Figure S10). Polymerization control remained excellent for all *E*_app_ values with low *Đ* and good agreement between experimental and theoretical molecular weights. The GPC traces shifted smoothly to higher MW (an example for *E*_app_ = *E*_1/2,ZnPC_ + 0.06 V is presented in Figure 3d).

**Table 3:** PET‐RAFT of BzMA under combined light irradiation and electric current. Effect of *E*_app_ and different target MWs (DP_target_).

Entry^a)^	DP_target_	*E*_app_ − *E*_1/2_ (mV)	*k*_p app_ (h^−1^)^b)^	*Conv* (%)^c)^	*M*_n_ ^d)^	*M*_n,th_	*I*_eff_ ^e)^	*Đ* ^d)^	*Q* (C)^f)^
1	100	0	0.33	72	14 400	13 100	0.91	1.18	−5.0
2	100	30	0.29	67	12 100	12 200	1.01	1.18	−3.9
3	100	60	0.19	61	10 600	11 000	1.04	1.17	−3.1
4	100	90	0.16	44	9160	8200	0.89	1.23	−2.2
5	200	30	0.31	69	25 700	24 700	0.96	1.16	−3.2
6	400	30	0.34	73	39 000	51 900	1.33	1.22	−2.6

^a)^ Polymerization conditions: BzMA in DMSO + 0.1 M *n*‐Bu_4_NBF_4_ at *T* = 50 °C, *V*_tot_ = 10 mL, reaction time 6 h; M = BzMA, CTA = CDTPA and Cat = ZnPC, [M]:[CTA]:[mediator] = DP_target_:1:0.02, at DP_target_ = 100, 200 and 400, respectively. Reaction time 6 h;

^b)^ The slope of the ln([M]_0_/[M]) versus time plot;

^c)^ Measured via ^1^H‐NMR;

^d)^ Measured via GPC with DMF eluent at 60 °C;

^e)^ *I*_eff_ = *M*_n,th_/*M*_n,GPC;_

^f)^ Measured from the chronoamperometry plot (see example in Figure S10).

### Different Target MW

The base‐promoted PET‐RAFT polymerization could produce polymers for different target degrees of polymerization (DP). Variation of the CTA amount to target DP in the range 100–400 resulted in nearly identical polymerization rates (Figure 3e), producing polymers in a range of MW from 9000 to 39 000, with good conversions and low *Đ* (Figure 3f).

### Chain End Livingness

**Figure 4:**
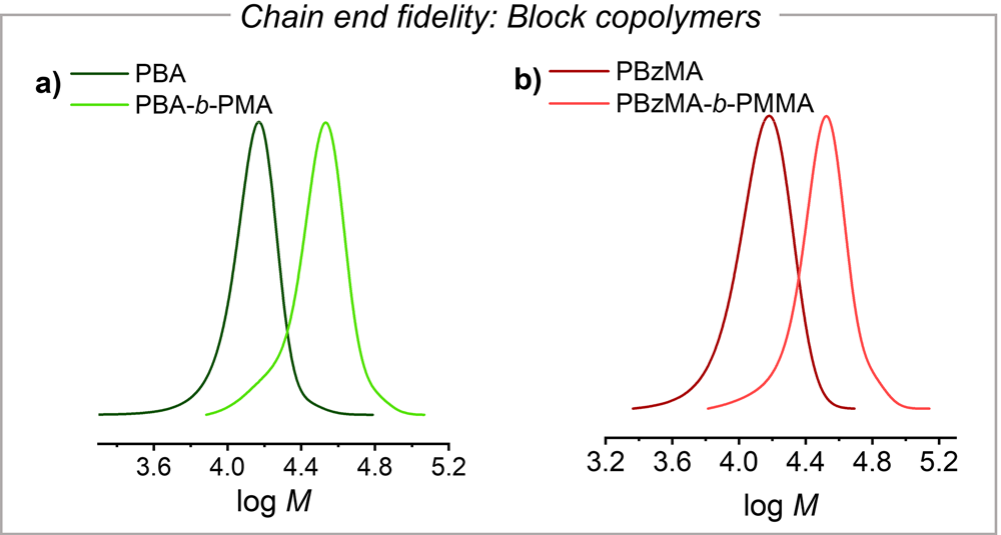
Chain extension experiments via a) photochemical RAFT and b) photo‐electrochemical RAFT. MW distribution of macroinitiator and block copolymer obtained by chain extension: a) PBA‐*b*‐PMA; b) PBzMA‐*b*‐PMMA.

To check the stability of the chain ends after PET‐RAFT with deprotonated CTAs, chain extension experiments were performed. A PBA macroCTA was prepared by the chemical deprotonation method: a BA solution was irradiated in the presence of ZnTPP, BTPA, and 0.3 equiv. *n*‐Bu_4_NOH relative to the CTA. After ca. 8 h of irradiation at 0.3 mW cm^−2^ the conversion reached ≥ 90%, with *M*_n_ = 13 550 and *Đ* = 1.10 (Figure 4a).

A second batch of monomer (methyl acrylate, MA) and solvent was added for an in situ chain extension. Already after 1 h, a clean shift to higher molecular weight was observed, producing a PBA‐*b*‐PMA block copolymer with *M*_n_ = 30 900 and *Đ* = 1.13. Reduced chain‐end fidelity was observed when the same reaction was carried out via the electrochemical deprotonation method (*E*_app_ = *E*_1/2,ZnTPP_ + 0.06 V), likely due to the direct reduction and subsequent decomposition of the CTA at the applied potential.

Excellent chain‐end fidelity was observed for the methacrylate polymers. A PBzMA‐*b*‐PMMA block copolymer was successfully prepared with the combined photo‐electrochemical approach. First, a PBzMA macroCTA was synthesized by irradiating a BzMA solution (0.3 mW cm^−2^) with ZnPC and CDTPA, under application of *E*_app_ = *E*_1/2,ZnPC_ + 30 mV. After 8 h, a PBzMA macroCTA with *M*_n_ = 16 200 at conversion ≥90% was obtained (Figure 4b). Addition of a methyl methacrylate (MMA) solution in DMSO under the same polymerization conditions triggered the in situ formation of a PBzMA‐*b*‐PMMA block copolymer with *M*_n_ = 32 050 and *Đ* = 1.14 after 15 h irradiation, with no tailing at low molecular weights, demonstrating excellent retention of chain‐end fidelity and catalyst activity. This chain extension experiments suggest that deprotonation of the CTA did not compromise its stability.^[^ ^8^ ^]^

### Mechanistic Studies

**Figure 5:**
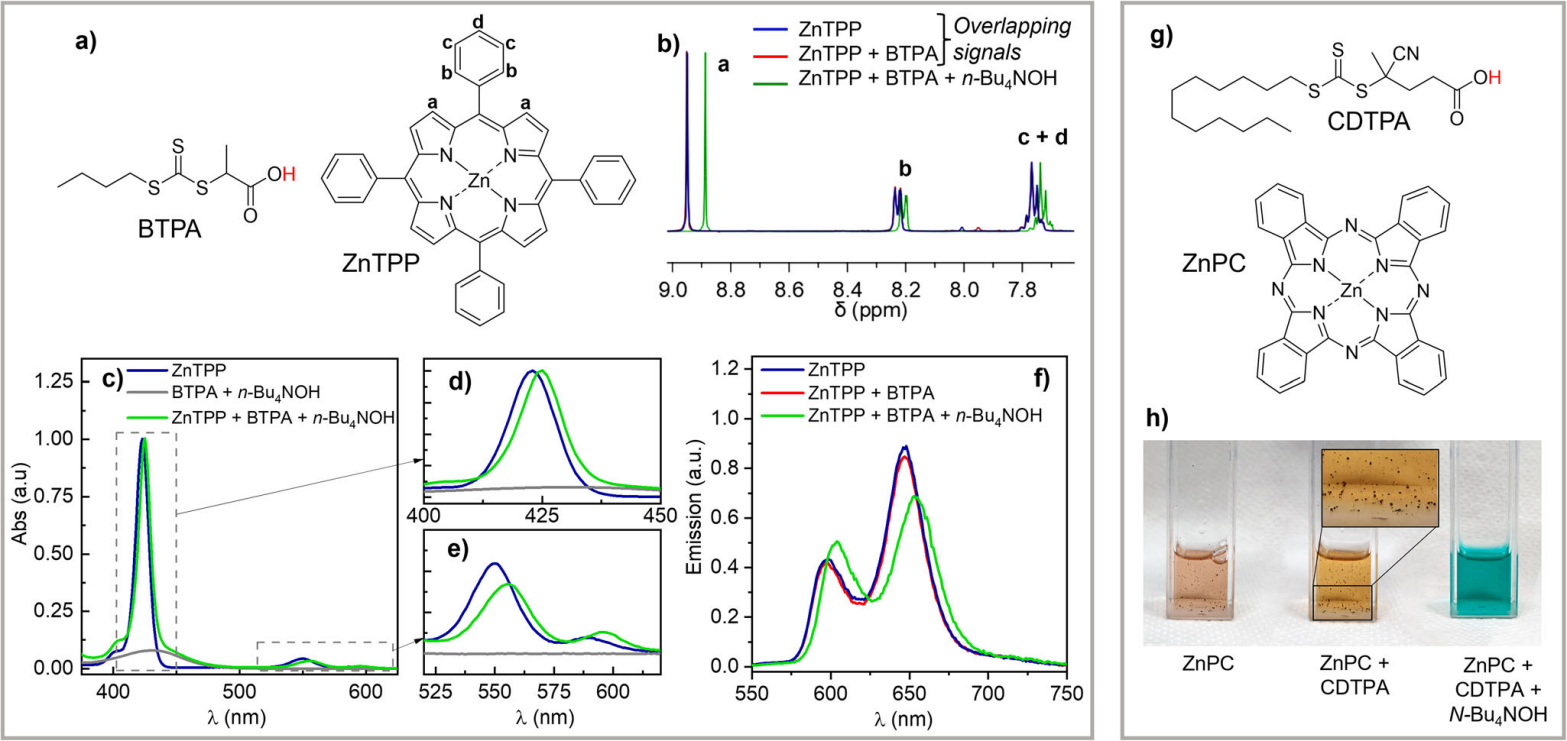
a) Chemical structures of BTPA and ZnTPP. b) ^1^H‐NMR of 10^−3^ M ZnTPP in CDCl_3_ in the absence and in the presence of 2 × 10^−3^ M BTPA or 2 × 10^−3^ M BTPA + 1.9 × 10^−3^ M n‐Bu_4_NOH. c) Intensity‐normalized UV‐vis spectra of 2 × 10^−2^ M BTPA + 1.9 × 10^−2^ M *n*‐Bu_4_NOH and of 10^−5^ M ZnTPP in toluene in the absence and in the presence of 2 × 10^−2^ M BTPA + 1.9 × 10^−2^ M *n*‐Bu_4_NOH. d) and e) Enlargements of the UV‐vis spectra in c) and f) Emission spectra of 10^−5^  M ZnTPP in toluene in the absence and in the presence of 2 × 10^−2^ M BTPA or 2 × 10^−2^ M BTPA + 1.9 × 10^−2^ M *n*‐Bu_4_NOH. Excitation wavelength = 540 nm. g) Chemical structures of CDTPA and ZnPC. h) Digital images of CDCl_3_ mixtures containing 10^−3^ M ZnPC in in the absence and in the presence of 2 × 10^−3^ CDTPA or 2 × 10^−3^ CDTPA + 2 × 10^−3^ *n*‐Bu_4_NOH.

To explore the increased RAFT reactivity under basic conditions, interactions between photocatalysts and deprotonated CTAs were examined. The ^1^H‐NMR spectra of ZnTPP were recorded both in the absence and in the presence of BTPA and *n*‐Bu_4_NOH. No change in the ¹H‐NMR spectra of ZnTPP was observed upon adding neat BTPA, as shown by the overlapping blue and red spectra in Figure 5b, indicating no interaction between the photocatalyst and the acidic CTA. Then, 0.95 equivalents of *n*‐Bu₄NOH relative to BTPA were added to deprotonate its carboxylic acid group. A substoichiometric amount of base was used to avoid forming (OH)ZnTPP⁻ complexes, leaving deprotonated BTPA as the only potential complexing agent. The presence of deprotonated BTPA altered the NMR spectra of ZnTPP, suggesting the formation of a complex between ZnTPP and the deprotonated CTA.^[^ ^30^ ^]^ A shift was also observed in the ^1^H‐NMR signals of BTPA (Figures S16–S19). Precomplexation was confirmed by UV–vis spectroscopy, where the spectra of ZnTPP in the presence of deprotonated CTA showed a significant shift (Figure 5c–e). The broad band at 430 nm was associated to the *n* → π* transition of the CTA.^[^ ^31^ ^]^

The emission spectra changed upon precomplexation (Figures 5f and S20), with the ZnTPP‐CTA complex showing weaker emission and shorter excited‐state lifetimes than bare ZnTPP (Figure S24). Both emission shape and lifetimes shifted progressively with increasing concentration of deprotonated CTA, stabilizing at 20 mM, likely due to the complete ZnTPP‐CTA complex formation in toluene. The reduced lifetime of the ZnTPP‐deprotonated BTPA precomplex suggests excited‐state reactivity that quenched fluorescence. In contrast, ZnTPP with fully protonated BTPA showed no changes in either spectra or lifetimes, indicating the absence of complexation (red line in Figure 5f).

The ZnTPP‐CTA complex showed much enhanced reactivity in PET‐RAFT, but was also emissive, as complete static quenching was not observed (Figure 5f). This indicates that the PET step does not efficiently compete with fluorescence. To further explore this aspect, thermodynamic and kinetic factors of the electron transfer between ZnTPP* and BTPA were investigated. The standard reduction potential of the photocatalyst $E_{{\mathrm{ZnTPP}}^{•+}/{\mathrm{ZnTPP}}^{*}}^{-o}$ was calculated by using the Rehm‐Weller relation as −1.72 V versus Fc^+^/Fc (se eq. S10), which is slightly less negative than the reduction potential of the CTA, observed at peak potential of −1.91 V versus Fc^+^/Fc. This suggests a slightly positive Gibbs free energy for PET, indicating inefficient electron transfer. Marcus theory was utilized to estimate the rate constant of electron transfer as *k*_PET_ ∼ 10^6^ s^−1^ (see eq. S11‐S14), which is about two orders of magnitude slower than the fluorescence rate (*k*_F_ = 1/*τ*_F_ = 5 × 10^8^ M^−1^ s^−1^). This explains the absence of static quenching, since the sluggish electron transfer is unable to efficiently compete with rapid fluorescence. Given the very high yield of triplet formation of ZnTPP, ca. 90%,^[^ ^32^ ^]^ most reactivity is expected to occur via electron transfer from this long‐lived triplet state.

Computational studies (COSMO‐ZORA‐B3LYP‐D3(BJ)/TZ2P//ZORA‐BP86/TZ2P)^[^ ^33^ ^]^ support the experimental precomplexation of ZnTPP with deprotonated BTPA. When CTAs are protonated, weak interactions with Zn via oxygen or sulfur are observed in toluene, but entropic effects destabilize these complexes by 7.3 and 2.7 kcal mol^−1^, respectively, preventing precomplexation. In contrast, deprotonated BTPA forms a stable complex with ZnTPP through a carboxylate oxygen, featuring a shorter Zn–O distance (2.0 Å vs 2.4 Å) and a slight displacement of the metal ion from the porphyrin plane. Including entropic corrections, the precomplex formation is nearly thermoneutral (−0.4 kcal mol^−1^), indicating weak coordination at equilibrium. This aligns with experimental findings that require a high excess (200X) of deprotonated BTPA for full complexation with ZnTPP in toluene. Similarly, coordination via sulfur shortens the Zn–S distance (2.6 Å vs 3.1 Å) with a reaction energy of −0.5 kcal mol^−1^.

UV‐Vis and ¹H‐NMR complexation experiments (Figures S21–S22) confirmed that ZnTPP forms a complex with deprotonated acetic acid (as tetraethylammonium acetate), indicating interaction between the carboxylate functionality and the Zn center. However, no interaction was detected between ZnTPP and neat acetic acid, neat BTPA, or the methyl ester of CDTPA, suggesting that neither the acid group nor the thiocarbonyl group binds ZnTPP (Figure S23). These results contrast with earlier studies that proposed coordination between the thiocarbonyl group of CTAs and ZnTPP.^[^ ^15^, ^34^ ^]^ Instead, our findings indicate the carboxylate group has stronger coordinating ability toward ZnTPP than the thiocarbonyl group. This aligns with prior observations of acid‐base interactions, such as coordination between the acidic group of CTAs and the pyrrole in pheophorbide A, as detected by NMR and UV‐vis spectroscopy.^[^ ^19^ ^]^ Control PET‐RAFTs at 0.25 mW cm^−^^2^ with ZnPC and nonacidic CTAs (cyanomethyl dodecyl trithiocarbonate, 2‐cyano‐2‐propyl dodecyl trithiocarbonate, and the methyl ester of CDTPA, Figure S5) under neutral conditions showed minimal to no conversion, highlighting the critical role of the carboxylate functionality in PET‐RAFT.

**Figure 6:**
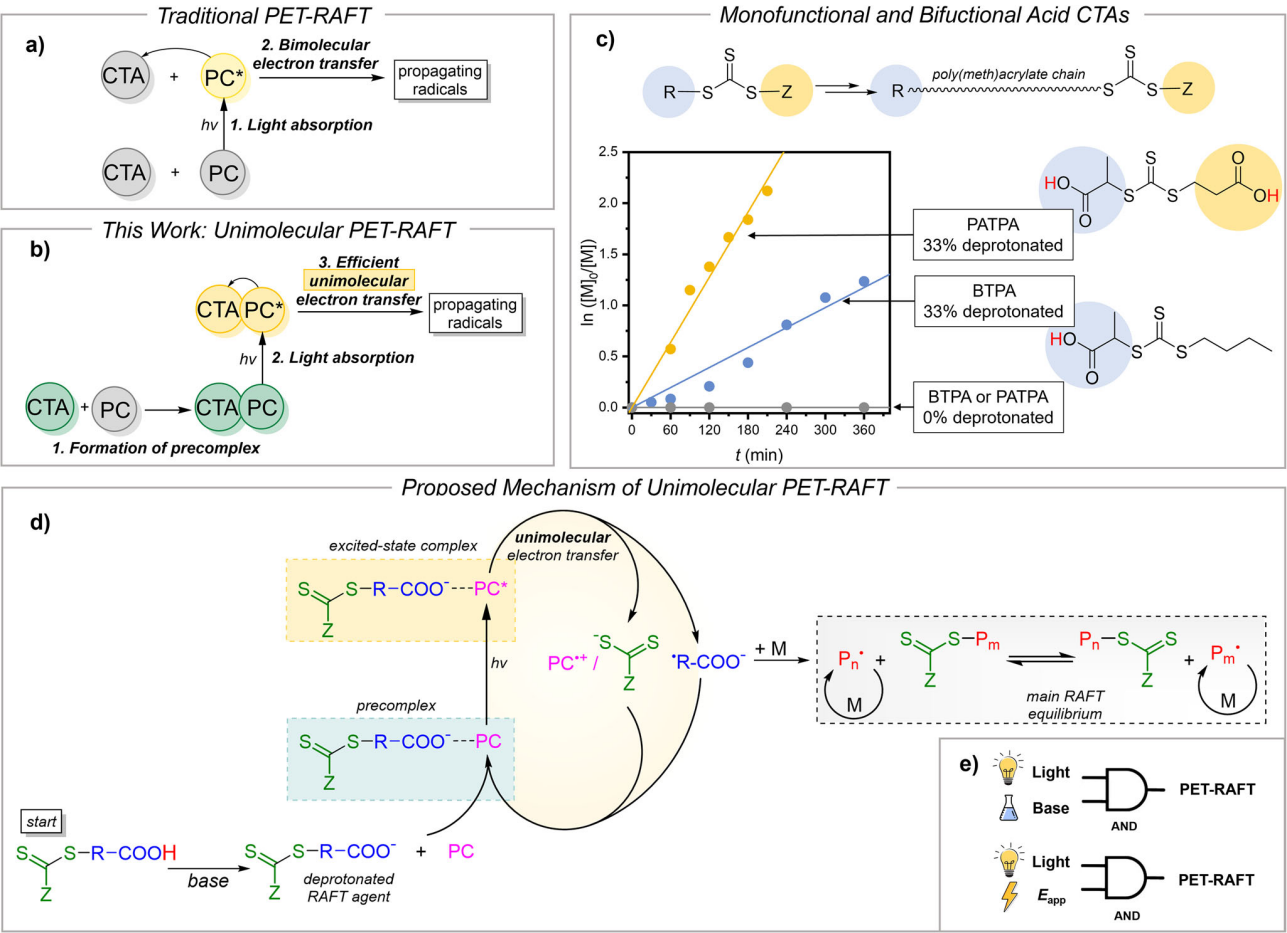
a) Simplified mechanism of traditional PET‐RAFT. b) Simplified mechanism of base‐enhanced unimolecular PET‐RAFT. c) PET‐RAFT polymerization with monofunctional acid (BTPA) and bifunctional acid (PATPA) as CTAs. Full polymerization conditions in Figure S28. d) Detailed proposed mechanism of PET‐RAFT polymerization via unimolecular activation of a CTA‐PC precomplex. e) Schematic of the logic‐gated PET‐RAFT polymerization concept (AND logic), either via chemical deprotonation (base) or electrochemical deprotonation (*E*_app_).

The formation of a PC−CTA precomplex likely shifts the mechanism from bimolecular electron transfer (Figure 6a) to more efficient unimolecular PET‐RAFT (Figure 6b). This increased reactivity is attributed to the proximity of reactive units in the ZnTPP‐deprotonated CTA precomplex, facilitating efficient electron transfer. Recently, precomplexation was also found to enhance the rate of photoinduced ATRP.^[^ ^35^ ^]^

Boyer et al. demonstrated enhanced polymerization rates by covalently tethering CTAs to free‐base porphyrins. In contrast, our system achieves similar proximity through acid‐base interactions rather than covalent bonding. A proposed mechanism (Figure 5d) suggests that base‐induced deprotonation of the CTA forms a PC−CTA precomplex, with the Zn center coordinating the carboxylate group. Weak light irradiation (*hν*) triggers unimolecular electron transfer from PC* to the bound CTA, leading to reductive cleavage and radical generation that drive RAFT polymerization.

The unimolecular activation mechanism was further optimized by replacing BTPA, which has a single carboxylic acid in the “R” group, with PATPA, containing carboxylic acid groups in both the “R” and “Z” positions (Figure 6c). Polymerization of BA with PATPA at 33% deprotonation was three times faster than with BTPA, while maintaining good polymerization control (Figure S28).

This rate enhancement likely stems from the strategic placement of carboxylic acid groups. In RAFT polymerization, the “R” group initiates the polymer chain and stays at its start, whereas the “Z” group moves with the growing polymer chain, remaining attached to the terminal thiocarbonyl group. With monoprotic BTPA (carboxylic acid in the “R” group), catalyst precomplexation primarily enhances the initiation phase, where the carboxylate group is near the C─S bond. This acceleration is significant since initiation can be rate‐limiting in RAFT systems.^[^ ^13^, ^36^ ^]^ Deprotonated PATPA, however, introduces an additional carboxylate group at the “Z” position, enabling catalyst complexation at the polymer's growing end. This “chain‐walking” mechanism allows the substoichiometric photocatalyst to remain attached to the chain end, ensuring efficient unimolecular PET‐RAFT activation throughout the polymerization. As a result, near‐complete monomer conversion was achieved in just 3.5 h, whereas fully protonated CTAs showed no polymerization under the same conditions (Figure 6c).

### Logic‐Gated Temporal Control

The unimolecular PET‐RAFT process could be controlled with an “AND” logic, capable of “processing” two external stimuli: both light and applied potential are required to enable polymerization (Figure 6e). Without either external stimulus, polymerization was nearly fully inhibited. Two ON/OFF experiments confirmed this behavior.

**Figure 7:**
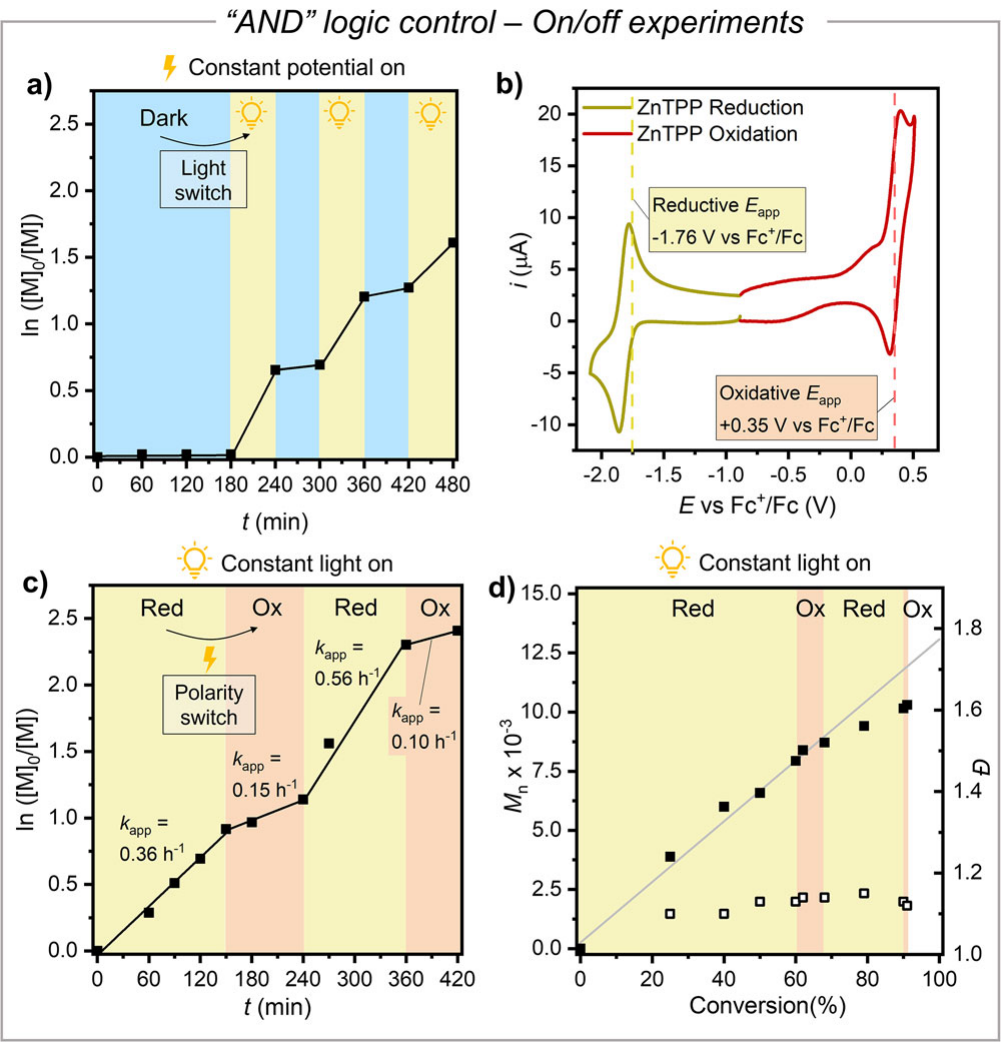
a) ON/OFF kinetic plots for PET‐RAFT polymerization of BA 50% (v/v) in DMSO using the catalytic system ZnTPP/BTPA, under constant *E*_app_ = −1.77 V versus Fc^+^/Fc. White light alternating between off and 0.3 mW cm^−2^. b) CV of 10^−3^ M ZnTPP in DMSO + 0.1 M *n*‐Bu_4_NBF_4_ recorded on a Pt disk electrode (*d* = 3 mm) at 0.1 V s^−1^ and *T* = 25 °C. c), and d) ON/OFF kinetics and MW plots for PET‐RAFT polymerization of BA 50% (v/v) in DMSO using the catalytic system ZnTPP/PATPA under continuous irradiation at 0.3 mW cm^−2^. Applied potential alternating between *E*_red_ = −1.76 V and *E*_ox_ = +0.35 V versus Fc^+^/Fc.

First, BA polymerization with BTPA/ZnTPP was tested under a constant cathodic potential. Polymerization occurred only when both light and cathodic potential were applied, with negligible growth during “light off” periods (Figure 7a).

Second, an electrochemical switch was tested under constant illumination (Figures 7b–d). In this case, polarity switching was necessary to affect polymerization, which proceeded under a reductive potential (*E*_app_ = −1.76 V vs Fc^+^/Fc) but significantly slowed upon switching to an oxidative potential after 150 min (*E*_app_ = 0.35 V vs Fc^+^/Fc). Restoring reductive conditions after 240 min resumed fast polymerization, which halted again after another “oxidative” switch at 360 min. The apparent propagation rate constant (*k*_app_) was on average four times lower during oxidative (OFF) periods. Polymerization remained well‐controlled, with low dispersity and good agreement between theoretical and experimental MWs. The halt under oxidative conditions may result from reversible CTA‐ZnTPP complex degradation, likely due to catalyst oxidation at positive potentials, occurring at a reversible wave with *E*_1/2_ = 0.35 V versus Fc^+^/Fc (Figure 7b).

### Chain Transfer Mechanism With Protonated and Deprotonated CTAs

**Figure 8:**
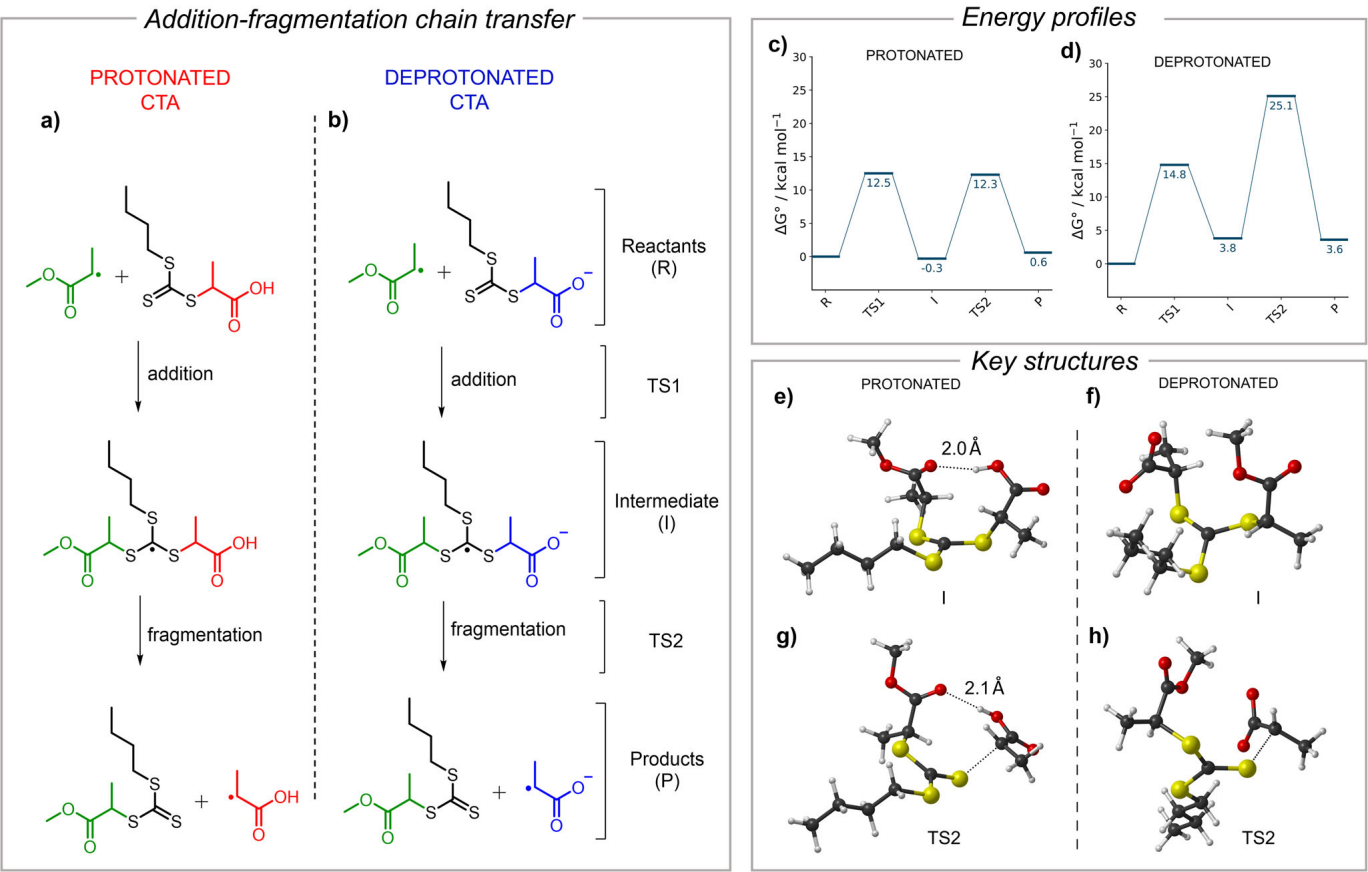
Model chain transfer reaction using methyl acrylate radical with a) protonated and b) deprotonated BTPA. Energy profile of the chain transfer reaction for c) protonated and d) deprotonated BTPA in DMSO. Gibbs free energies are reported in kcal mol^−1^ with respect to the free reagents. Fully optimized structures of the intermediate (I) for the e) protonated and f) deprotonated BTPA. Fully optimized structures for the TS2 transition state of the chain transfer mechanism for the g) protonated and h) deprotonated BTPA. Level of theory: COSMO‐ZORA‐M06‐2X/TZ2P//ZORA‐OLYP/TZ2P.

Partial deprotonation of CTAs enabled efficient PET‐RAFT polymerization with excellent control, while full deprotonation reduced control by accelerating PET but slowing chain transfer (Figure 1b,d). Thermal RAFT polymerization confirmed slower chain transfer for fully deprotonated CTAs in BTPA/acrylate and CDTPA/methacrylate systems (Table S2). This sluggish transfer arises from either delayed radical addition to the CTA (TS1 in Figure 7a) or slow fragmentation of the radical intermediate (TS2 in Figure 8a), though pinpointing the rate‐determining step experimentally is challenging.

To investigate this, computational analysis was performed modeling a methyl acrylate radical adding to BTPA, followed by release of the acid or carboxylate group through homolytic C─S bond cleavage (Figure 8a). Gibbs free energy profiles (COSMO‐ZORA‐M06‐2X/TZ2P//ZORA‐OLYP/TZ2P)^[^ ^37^, ^38^, ^39^, ^40^, ^41^ ^]^ in DMSO revealed key differences between protonated and deprotonated systems. For protonated BTPA, radical addition is thermoneutral (Δ*G* = −0.3 kcal mol^−1^), with a 12.5 kcal mol^−1^ barrier (TS1) forming a stabilized intermediate. Fragmentation requires 12.6 kcal mol^−1^ (TS2) and is nearly thermoneutral (Δ*G* = 0.6 kcal mol^−1^), thus maintaining the equilibrium typical of RAFT systems.

In contrast, deprotonated BTPA requires higher activation energies. Radical addition has a 14.8 kcal mol^−1^ barrier, and the intermediate (I) lies 3.8 kcal mol^−1^ higher. Fragmentation faces a steep 21.3 kcal mol^−1^ barrier, with a reaction energy of 3.6 kcal mol^−1^. Deprotonation disrupts the RAFT exchange by hindering fragment exchange and raising activation energies, particularly in the fragmentation step.

Structural differences explain this disparity. In protonated BTPA, the intermediate radical is stabilized by an intramolecular hydrogen bond between the acidic proton and ester oxygen (Figure 8e), absent in the deprotonated form (Figure 8f). This stabilization lowers the energy of TS2 (Figure 8g), facilitating fragmentation. Without this hydrogen bond, deprotonated BTPA experiences a higher TS2 energy, reducing chain transfer efficiency and polymerization control.

## Conclusions

In this study, a more efficient approach to photoinduced RAFT polymerization was developed. Deprotonation of common acidic CTAs enhanced polymerization efficiency under low‐intensity light irradiation, enabling polymerization to occur under ambient light conditions or with power levels as low as 0.25 mW cm^−^^2^. This methodology was compatible with both acrylates and methacrylates and allowed for the preparation of well‐defined block copolymers. The efficiency improvement stems from precomplex formation between the photocatalyst and the CTA, enabling a shift from bimolecular to unimolecular electron transfer mechanism.

Specifically, the ZnTPP‐BTPA, ZnTPP‐CDTPA, and ZnPC‐CDTPA systems exhibited fast polymerization and good molecular weight control with partial CTA deprotonation, while full deprotonation led to diminished control due to slower chain transfer.

Using of a CTA with carboxylate functionality in both the R and Z groups (PATPA) further accelerated the polymerization rate by threefold, thanks to “chain‐walking” of the photocatalyst bound to the carboxylate chain end of the polymer.

This work introduced a novel externally gated polymerization using a combined photo‐electrochemical approach, requiring both light and an applied potential to drive polymerization (“AND” logic). Notably, reversing the electrolysis polarity nearly halted the photopolymerization rate. This dual‐stimulus strategy enabled precise and reversible control over the polymerization. The voltammetric studies elucidated the electrochemical behavior of the photocatalysts and their ability to facilitate the deprotonation of the CTAs under reducing conditions, while oxidative conditions could reversibly halt the polymerization.

Further improvement of this method could involve the reduction of catalyst loading, and the exploration of hydrophobic solvents, which we found challenging for the BA/ZnTPP system. Overall, the development of base‐enhanced PET‐RAFT polymerization offers an efficient and scalable method for synthesizing well‐defined polymers under mild conditions (weak light irradiation combined with partial CTA deprotonation). This method holds great potential for applications where energy efficiency and precise control over polymer architecture are critical.

## Conflict of Interests

The authors declare no conflict of interest.

## Supporting information



Supporting Information

## Data Availability

The data that support the findings of this study are available from the corresponding author upon reasonable request.
