# Supplementary material for: Photo‐RAFT Polymerization Under Microwatt Irradiation via Unimolecular Photoinduced Electron Transfer
Source: Angew Chem Int Ed Engl. 2025 Mar 17;64(19):e202424225. doi: 10.1002/anie.202424225 (PMC12051724; doi:10.1002/anie.202424225)
Supplement: Supplementary file 1 — Supporting Information [file ANIE-64-e202424225-s001.docx]

Supporting Information for

**Photo-RAFT Polymerization under Microwatt Irradiation via Unimolecular Photoinduced Electron Transfer**

Giovanni Lissandrini,^a^ Davide Zeppilli,^a^ Francesca Lorandi,^b^ Krzysztof Matyjaszewski,^c^ Abdirisak A. Isse*,^a^ Laura Orian,^a^ Marco Fantin^a^*

^a^Department of Chemical Sciences, University of Padova, Via Marzolo 1, 35131, Padova, Italy

^b^Laboratory for Macromolecular and Organic Chemistry, Department of Chemical Sciences, University of Padova, 35131 Padova, Italy

^c^Department of Chemistry, Carnegie Mellon University, Pittsburgh, Pennsylvania 15213, United States

Corresponding authors: [marco.fantin@unipd.it](mailto:marco.fantin@unipd.it); [abdirisak.ahmedisse@unipd.it](mailto:abdirisak.ahmedisse@unipd.it)

**Table of Contents**

[Materials and methods 2](#_Toc183336901)

[Procedures 4](#_Toc183336902)

[Additional data for PET-RAFT with protonated and deprotonated CTA 8](#_Toc183336903)

[Additional data for externally gated PET-RAFT by a combined photo-electrochemical approach 9](#_Toc183336904)

[Characterization of the electrolysis products 14](#_Toc183336905)

[Additional spectroscopic data 16](#_Toc183336906)

[Thermodynamic aspects of photoinduced electron transfer 22](#_Toc183336907)

[Computational methods 25](#_Toc183336908)

[References 35](#_Toc183336909)

# Materials and methods

***Materials***

Methyl acrylate (MA, 99% Sigma Aldrich), methyl methacrylate (MMA, 99% Sigma Aldrich), butyl acrylate (BA, 99% Sigma Aldrich), and benzyl methacrylate (BzMA, 99% Sigma Aldrich) were passed in a column filled with basic alumina (Sigma Aldrich) to remove the inhibitors. Dimethyl sulfoxide (DMSO, 99.9% Fisher Scientific), HPLC grade *N,N*-dimethylformamide (DMF, 99.9% Carlo Erba), acetonitrile (MeCN, 99.8% Sigma Aldrich), sulfuric acid (95-98% Sigma Aldrich), and deuterated chloroform (CDCl_3_, 99.8% Sigma Aldrich) were used as received. The supporting electrolyte, tetrabutylammonium tetrafluoroborate (*n*-Bu_4_NBF_4_, Alfa Aesar, 98%), was recrystallized twice from hot ethyl acetate and dried in a vacuum oven at 80 °C, over a weekend, and then stored over P_2_O_5_. 4-cyano-4-[(dodecylsulfanylthiocarbonyl)sulfanyl]pentanoic acid (CDTPA, 97% Boron Molecular), 2-(butylthiocarbonothioylthio)propanoic acid (BTPA, 95% Boron Molecular), cyanomethyl dodecyl trithiocarbonate (CDT, 97% Sigma Aldrich), 2-cyanobutan-2-yl dodecyl carbonotrithioate (CDTB 97% Sigma-Aldrich), methyl 4-cyano-4-(dodecylthiocarbonothioylthio)pentanoate (CDTPE, 97%, Boron Molecular), 3-((((1-carboxyethyl)thio)carbonothioyl)thio)propanoic acid (PATPA, 95%, Boron Molecular), Zinc tetraphenylporphyrin (ZnTPP, 99% TCI Chemicals), zinc phthalocyanine (ZnPC, 99% TCI Chemicals), tetrabutylammonium hydroxide solution (*n*-Bu_4_NOH, 1 M in methanol, Sigma Aldrich), and ferrocene (Fc, 98% Acros Organics) were used as received. 2,2'-Azobisisobutyronitrile (AIBN, 98% Sigma Aldrich) was recrystallized using absolute ethanol and dried in a vacuum oven at 25 °C, over a weekend and stored over P_2_O_5_. All the other reagents were of high commercial grade and used without further purification.

Instrumentation

*Gel permeation chromatography.* GPC was carried out using an Agilent 1260 Infinity to determine the average molecular weights and dispersity of the polymers synthesized through the RAFT technique. The instrument was equipped with two PSS GRAM columns, each sized at 300 mm × 10 mm and a universal detector with a refractive index detector (RID). The system also included an on-line degasser. For polymer sample analysis, an eluent consisting of a 10 mM LiBr solution in DMF was used, with a flow rate of 1 mL/min. The column temperature was maintained at 60 °C, while the RID temperature was set at 50 °C. Column calibration was performed using 12 standard samples of linear polymethylmethacrylate (PMMA) provided by Agilent EasiVial, with average molecular weights (*M*_n_) ranging from 540 to 2,210,000. Before injection (100 µL), the samples were filtered through a polytetrafluoroethylene (PTFE) membrane with a 200 nm pore size.

*Nuclear Magnetic Resonance Spectroscopy.* 400 MHz ^1^H-NMR spectra of reaction mixture samples in CDCl_3_ with tetramethylsilane (TMS) as an NMR internal standard were recorded on a Bruker Avance III 400 MHz spectrometer.

*UV–Vis-NIR*. Spectroscopy was performed with an Agilent UV–vis-NIR Cary 5000 Spectrometer.

*Fluorescence Spectrometer.* For fluorescence measurements, the FLS 1000 UV/Vis/NIR photoluminescence spectrometer (Edinburgh Instruments Ltd.) was used. The excitation source was a 450W Xenon arc lamp that emits continuous radiation from 230 nm to over 1000 nm. The detector used was an air-cooled single-photon counting photomultiplier (Hamamatsu R13456). This is a side-window photomultiplier with extended sensitivity in the near-infrared range, covering a spectral range from 185 nm to 980 nm.

For time-resolved lifetime measurements, the Time-Correlated Single Photon Counting (TCSPC) technique was used. TCSPC is a digital technique for detecting single photons with a time resolution of less than 5 ps. The sample was excited by a high-frequency pulsed light source (laser diode at 402.6 nm). With a typical repetition rate of 10^5^-10^7^ cps, a probability histogram of photon arrival times is quickly generated. Decay curves were analyzed with the Fluoracle Software using the IRF convolution and one-exponential component model fitting.

*Electrochemistry.* Electrochemical experiments were carried out with an Autolab potentiostat (PGSTAT30, Utrecht, The Netherlands) run by a computer with GPES software. All experiments were performed in a jacketed 6-neck glass electrochemical cell. The electrodes used in cyclic voltammetry were a platinum (Pt) or a glassy carbon (GC) disk electrode as the working electrode, a platinum mesh electrode as the counter electrode, and an Ag|AgI|I^-^ reference electrode prepared by dipping an AgI-coated Ag wire in 0.1 M *n*-Bu_4_NI in DMF. The reference electrode was calibrated against the ferrocenium/ferrocene couple after each experiment. During electrolysis, a platinum mesh electrode was used as the working electrode together with the Ag|AgI|I^-^ reference electrode and a graphite counter electrode in a separate compartment containing the same electrolyte of the cathode compartment (0.1 M di *n*-Bu_4_NBF_4_ in DMF). Before its first use, the electrochemical cell was cleaned with *aqua regia* (**caution! This is a highly corrosive and oxidizing cleaning solution**), and abundantly washed with ultrapure water from Milli-Q system followed by HPLC grade acetone, and dried in an oven at ~60 °C.

# Procedures

*Activation of Pt mesh working electrode.* Before each electrolysis, the Pt mesh was subjected to a cleaning and electrochemical activation process through approximately 50 voltammetric scans at a scan rate of 0.2 V/s and a potential range between -0.7 V and +1.0 V vs Hg/Hg_2_SO_4_ in 0.5 M H_2_SO_4_. Subsequently, it was rinsed with ample water, then acetone and dried.

*Cyclic voltammetry*. For the voltammetric investigations, two types of working electrodes were employed: a glassy carbon disk (GC, *d* = 3 cm, Tokai) and a platinum disk (Pt, *d* = 3 cm, Metrohm). To ensure data reproducibility and correct electrochemical performance, the electrodes were subjected to a thorough cleaning if they have been inactive for an extended period or exhibited passivation phenomena. For daily use, a less rigorous cleaning procedure was employed. For thorough cleaning, they were first polished to a mirror finish by using silicon carbide papers (1000, 2000 and 4000 grit), followed by diamond pastes of decreasing grain size of 3, 1, and 0.25 μm on Buelher® cloths; they were sonicated in absolute ethanol for five minutes after each polishing, and finally rinsed with HPLC acetone. The routine cleaning, instead, involves a single abrasion step using diamond paste with 0.25 μm particles, followed by sonication in ethanol and rinsing with HPLC acetone. The electrochemical cell (Figure S1) was loaded with 0.1 M of the supporting electrolyte in 10 mL of solution. The mixture was stirred and purged with Argon for 30 min before the experiment. After that time, CVs were recorded.


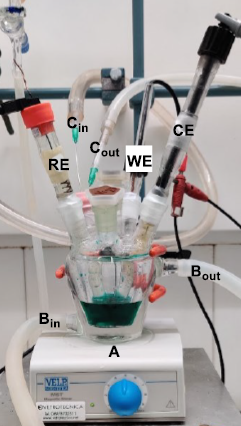


**Figure S1**. Electrochemical cell setup showing electrodes, magnetic stirrer (A), water circulation for temperature control (B), and inert gas line for degassing (C).


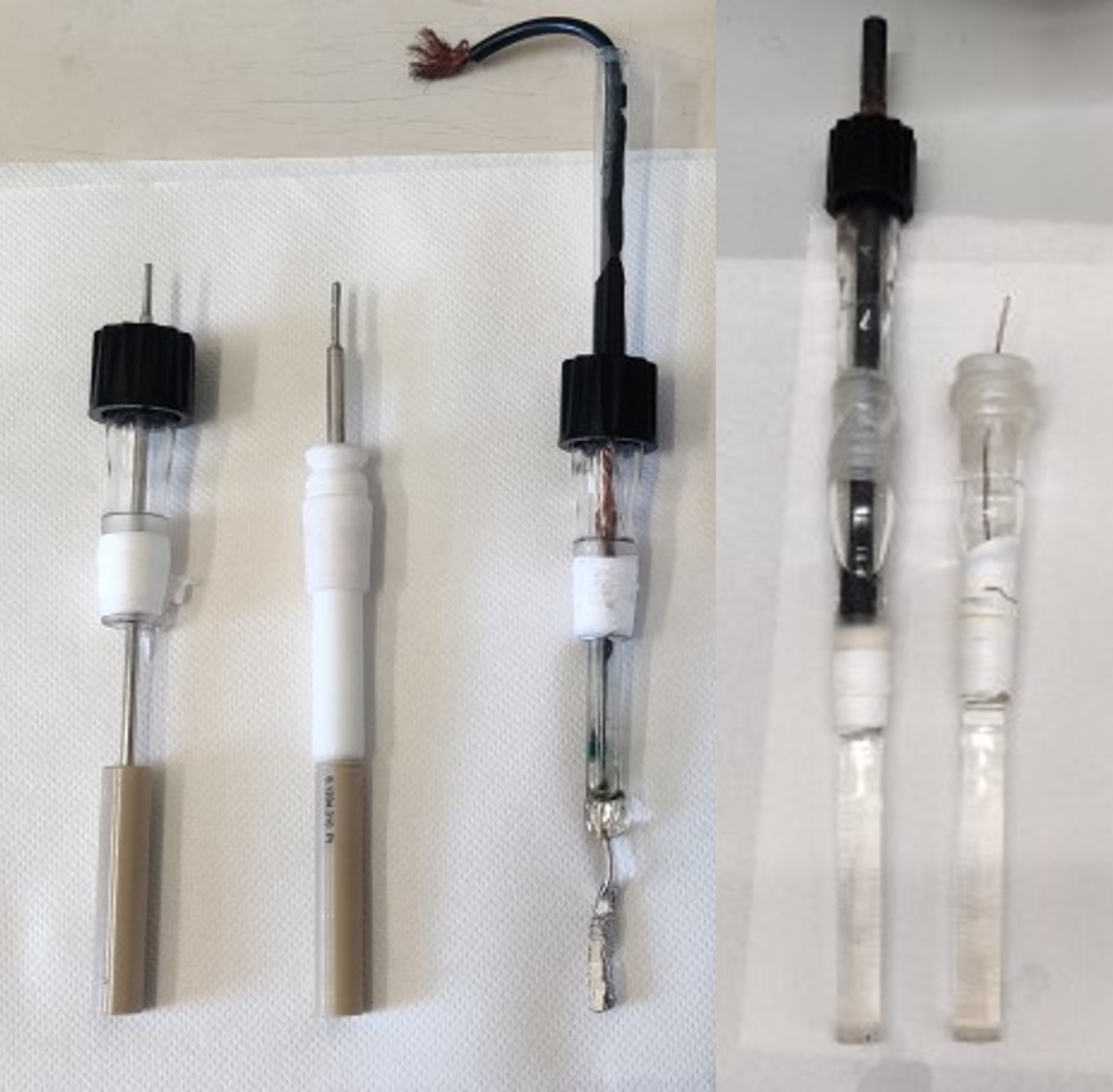


**Figure S2.** From left to right: GC disk, Pt disk, Pt mesh, graphite CE and RE Ag|AgI|I^-^.

*General procedure for electrochemical RAFT polymerization.* The electrochemical cell, equipped with the electrodes described earlier (see electrolysis section) and under a flow of Ar, was covered with a black fabric to avoid any light irradiation, and thermostated at *T* = 50 °C. The supporting electrolyte (0.32g, 0.1 M), monomer (50 vol%) and solvent (DMSO) were added into the cell, and a background CV was recorded (*V*_tot_ = 10 mL). The mediator and RAFT agent were inserted into the cell (for ZnPC, 3.4 mg (0.6 mM) were added, and for CDTPA, 0.119 g (0.03 M) were added; for ZnTPP, 4.4 mg (0.6 mM) were added, and for BTPA, 0.0781 g (0.03 M) were added) and CVs were recorded after every addition. The solution was further degassed for 20 minutes with a stream of Ar. A selected potential *E*_app_ was then applied, and the current was monitored during electrolysis. Samples were periodically withdrawn with a syringe to follow polymerization kinetics by ^1^H NMR and to characterize the polymer by GPC. When the polymerizations were stopped, CVs were recorded, then ferrocene was added, and the potential of Fc^+^/Fc couple was measured by CV. When polymerizing acrylates (MA and BA), the electrochemical cell was equipped with a condenser to prevent monomer evaporation (Figure S3, right).

*General procedure for RAFT polymerization under combined photo-electrochemical approach.* The same setup and procedure as described above for the electrochemical RAFT polymerization was utilized. Additionally, the cell was illuminated from one side with a white light LED strip of 6 units (6000 K, SMD 2835, Lepro) placed at a 10 cm distance, which were measured to output 0.25 mW/cm^2^ in the center of the reaction vessel (see below).

| 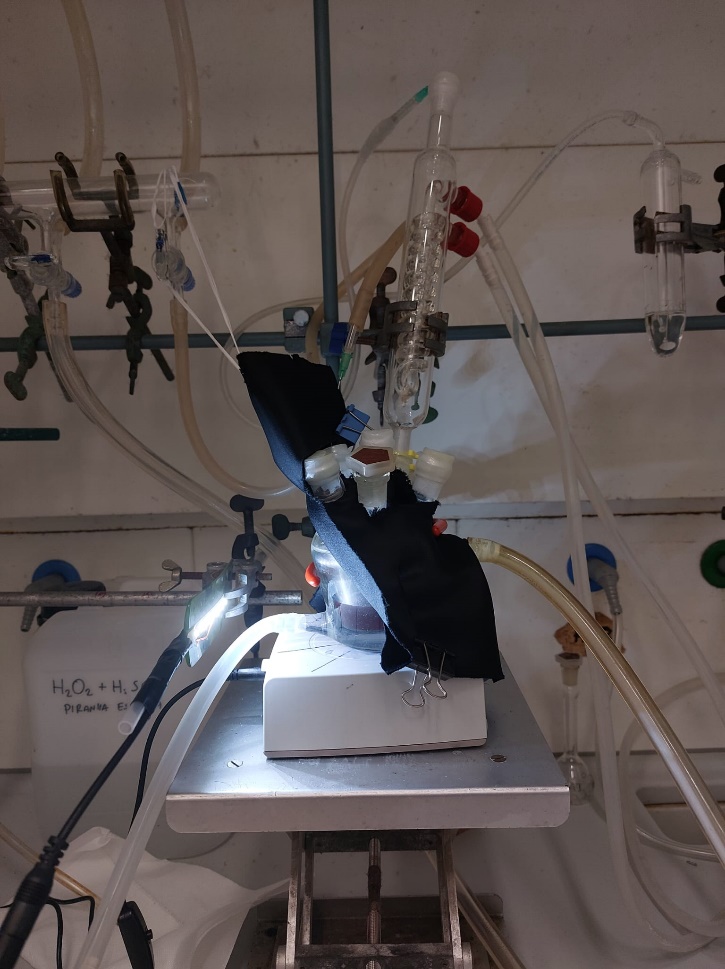 | 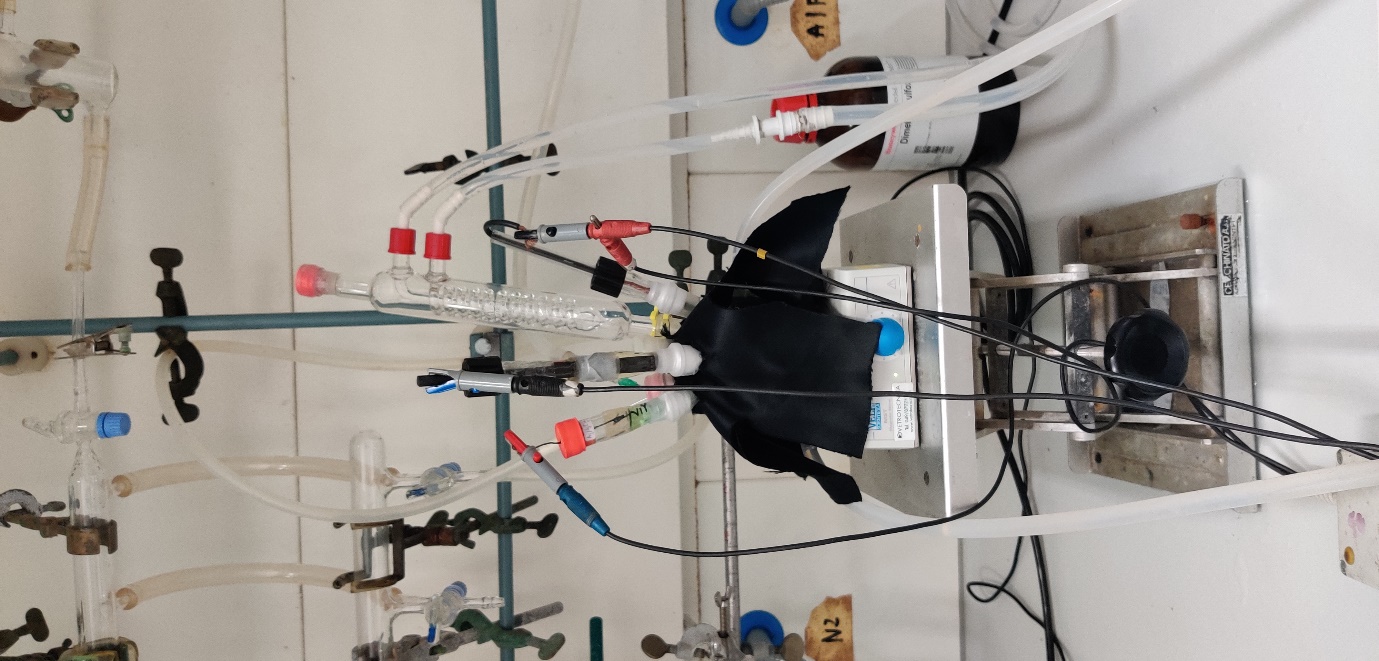 |
| --- | --- |

**Figure S3.** (left) Setup during photochemical or combined photochemical-electrochemical experiments. (Right) Setup during electrochemistry-only experiments, with light shielding.

*General procedure for photoinduced RAFT polymerization.* To have a meaningful comparison between polymerizations, photochemical reactions were carried out in an electrochemical cell but without the application of any potential. The reaction was thermostated at 50 °C. The same light source was used, comprising of a strip of 6 white light LEDs (6000 K, SMD 2835, Lepro) placed at a 10 cm distance, which were measured to output 0.25 mW/cm^2^ (Figure S3, right).

*Procedure for conventional RAFT polymerization.* The conventional RAFT polymerization was also conducted in an electrochemical cell, in the presence of supporting electrolyte, to use the same setup described previously. The cell was placed under argon degassing and thermostated at 60 °C. Monomer (50 vol%) and solvent (DMSO) were added into the cell (*V*_tot_ = 10 mL), followed by the chain transfer agent (CTA) and 10% AIBN initiator (2.68 mg, 1.6 mM); BTPA (0.038 g, 0.016 M) and CDTPA (0.059 g, 0.014 M) were used as CTA for acrylate and methacrylate polymerizations, respectively.

*Chain extension and block copolymer synthesis via RAFT polymerization.* For the synthesis of acrylate and methacrylate block copolymers, the same setup described previously for RAFT polymerization was employed. The process was conducted in a 50:50 (vol) mixture of DMSO and M, with a target DP of 100, achieving high monomer conversion (>85%). The first polymer block was synthesized using various approaches (as previously described, with *V*_tot_ = 10 mL). Subsequently, the second polymer block was synthesized following the same approach as the first block but with a target DP of 200 to observe significant shifts in the GPC curves. During the synthesis of the second polymer block, no additional catalyst or CTA was added. Five milliliters of monomer were used in 15 mL of solvent to ensure excellent solubility and mixing. The monomer/solvent mixture was degassed with argon for 30 minutes in a 20 mL vial, then withdrawn with a syringe (under argon) and inserted into the electrochemical cell containing the first polymer block. Samples were periodically withdrawn with a syringe to monitor the kinetics via ^1^H NMR and to characterize the polymer via GPC.

*Characterization of the light setup.* The irradiation power of the LED light (6 LED units taken from a 6000 K, SMD 2835, Lepro white light LED strip, Figure S4) was measured with an AvaSpec-2048 Fiber Optic Spectrometer from Avantes. The LEDs were placed at 10 cm from the probe, and an irradiance of 0.25 mW cm^-2^ was measured. For comparison, the light at the bottom of the hood where the electrochemical cell is located (Figure S3), coming from LED light on top of the hood, was measured at a similar intensity of 0.25 mW/cm^2^. A comparison of the irradiation spectrum of the LED lights with that of the laboratory light (bottom of the hood) is shown in Figure S4.


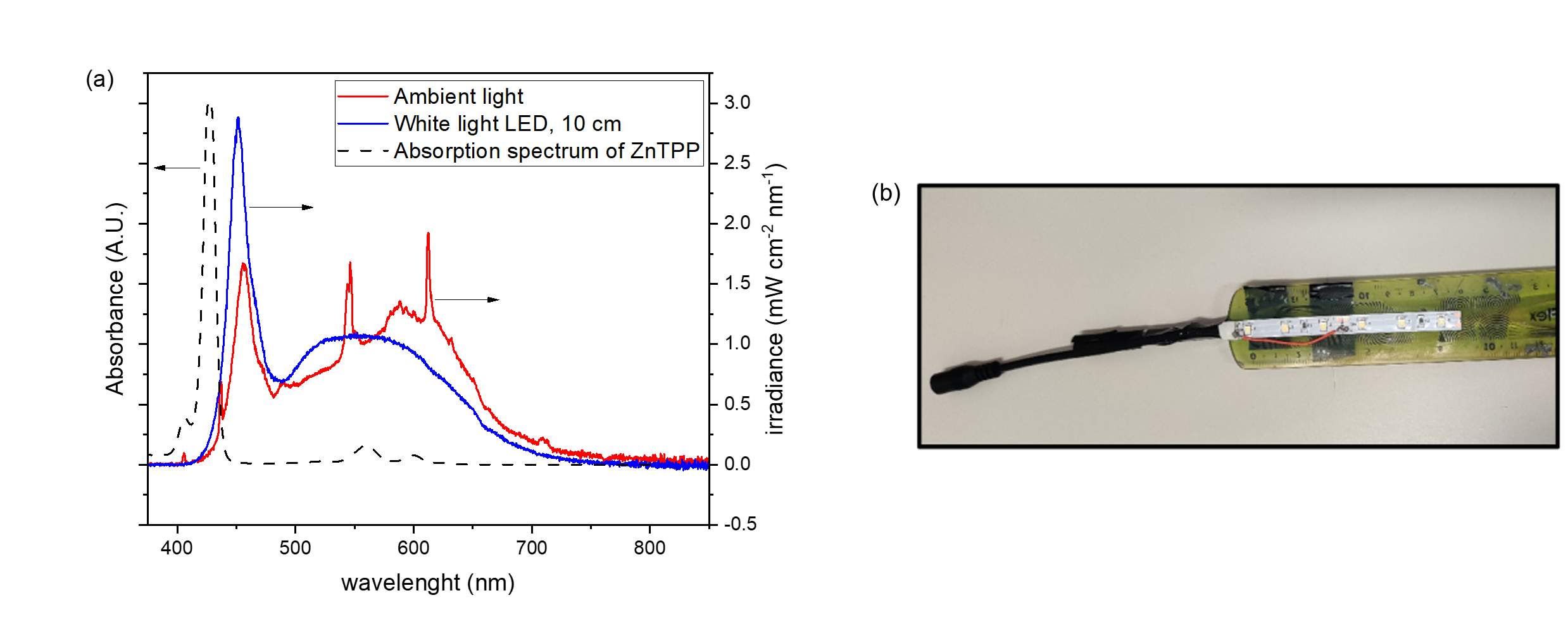


**Figure S4.** a) Irradiance of white light LED (10 cm from reactor), ambient light (measured at the bottom of a lab hood). Absorption spectrum of ZnTPP in DMSO/BA 50% vol is added to the graph for reference. b) Digital Picture of the LED strip.

#

# Additional data for PET-RAFT with protonated and deprotonated CTA

*Control experiments of PET-RAFT polymerization.*

**Table S1.** Control polymerization experiments of BA and BzMA.

| *Entry^a^* | *monomer* | *CTA* | *% deprotonation* | *PC* | *[M]:[CTA]* | *[M]:[CTA]* | *Conv (%)* |
| --- | --- | --- | --- | --- | --- | --- | --- |
| 1 | BA | BTPA | 33 % | None | 100:1 | 3.27:0.032 | <5 |
| 2 | BzMA | CDTPA | 33 % | None | 100:1 | 2.95:0.029 | <5 |
| 3^b^ | BA | BTPA | 33 % | ZnTPP | 100:1 | 3.27:0.032 | <5 |
| 4 | BA | none | 33 % | ZnTPP | 100:1 | 3.27:0 | <5 |

^a^ General polymerization conditions: *T* = 50 °C, 50% (v/v) M in DMSO, *V*_tot_ = 10 mL, 0.25 mW/cm^2^ LED positioned 10 cm away from the reactor. Reaction time = 6 h. ^b^No light, also excluding any ambient light.

*Effect of CTA Deprotonation in RAFT with Thermal Activation.* These experiments aimed to understand thermally activated RAFT polymerization using AIBN with protonated or deprotonated CTA, excluding light. The goal was to see if complete deprotonation of the CTA causes loss of control over the reaction, leading to polymers with unexpected molecular weights. Four thermal activation experiments using AIBN were conducted without monitoring the kinetics due to oxygen sensitivity; samples were analyzed at the end via GPC and NMR. Two experiments were for acrylates (BA)—one with non-deprotonated and one with fully deprotonated CTA using *n*-Bu_4_NOH—and two identical experiments for methacrylates (BzMA). The results (Table S2) showed that deprotonation of CTA reduces control over polymerization, resulting in significantly higher molecular weights than expected.

**Table S2.** Thermal RAFT polymerizations in DMSO, using AIBN as the initiator at 10 mol% relative to the CTA.

| *Entry^a^* | *[M](mol/L) ^b^* | *% CTA deprotonated ^c^* | *Conv (%)* | *M_n_ ^d^* | *M_n,th_ ^e^* | *Ð^d^* |
| --- | --- | --- | --- | --- | --- | --- |
| 1^f^ | 3.27 | 0 | 70 | 17900 | 18200 | 1.10 |
| 2^f^ | 3.27 | 100 | 57 | 61800 | 14900 | 1.97 |
| 3^g^ | 2.95 | 0 | 54 | 17500 | 19400 | 1.31 |
| 4^g^ | 2.95 | 100 | 42 | 30200 | 15200 | 2.22 |

^a^General polymerization conditions: *T* = 60 °C, 50% (v/v) M in DMSO, *V*_tot_ = 10 mL, reaction time 5 h. ^b^[M]:[CTA]:[AIBN]= 100:0.5:0.05. ^c^Via addition of *n-*Bu_4_NOH. ^d^Measured via GPC with DMF eluent at 60 °C. ^e^*M*_n,th_ = *M*_w,CTA_ + *M*_w,M_ × DP_target_ × % conv. ^f^M = BA, ^g^M = BzMA.

*RAFT polymerization with CTAs without carboxylic functionality.*

These control experiments were aimed to demonstrate that deprotonation of the carboxylic function of the CTA, whether through electrochemical or chemical processes, is essential for the generation of the radicals needed to initiate the polymerization reaction, thanks to the formation of a complex with the photocatalyst. To this end, we used three CTAs devoid of the carboxylic group (Figure S13).

**Figure S5.** The structure of CDT, CDTB, and CDTPE.

The selected CTAs are very similar to the RAFT agent CDTPA used for BzMA, as the Z group is a hydrocarbon chain with C_12_ atoms. The main difference lies in the R group, which does not contain the carboxylic group and generates a primary radical (CDT) or a tertiary radical (CDTB and CDTPE). The general procedure for PET-RAFT polymerizations was followed. No polymerization of BzMA was observed with these CTAs and ZnTPP.

# Additional data for externally gated polymerization by a combined photo-electrochemical approach

*CV of RAFT agents.* The voltammetric behavior of CTA presents interpretative challenges. Typically, the electrochemical reduction of RAFT agents exhibits two well-defined regions, referred to as region A and B [Figure S6].


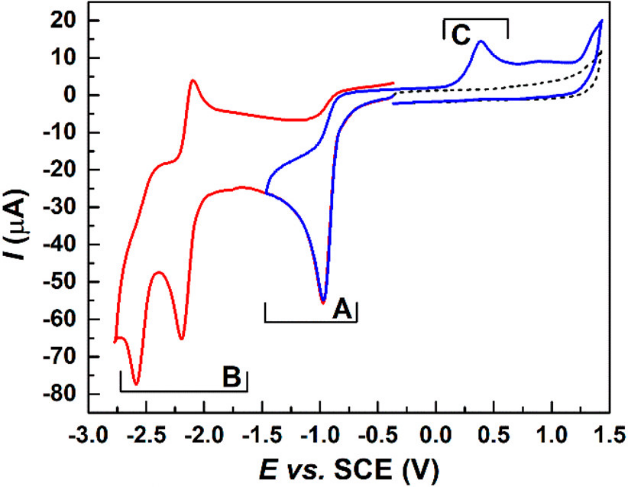


***Figure S6.*** *Typical CV of a RAFT agent. The letters A, B, and C denote the regions described in the text below and are common to all RAFT agents. The dashed line represents an oxidative scan not preceded by the reduction of the CTA. Obtained from reference.*^1^

Region A represents the first reduction of the RAFT agent, leading to the formation of the corresponding radical anion which then undergoes fragmentation.

When extending the potential range to more negative values, various voltammetric peaks involving different reactions and products are observed, indicating a complex reduction mechanism. It is important to note that the electrogenerated radical anion is not stable within the timescale of cyclic voltammetry, and reversing the potential scan does not allow the reoxidation of the radical anion to the initial compound, resulting in irreversible voltammetry.

***Figure S7.*** *Voltammetric behavior upon different additions of n-Bu_4_NOH of the CTAs in DMSO + 0.1 M Bu_4_NBF_4_ on a GC electrode at v = 0.1 V/s and T = 30 °C. a) 2 mM BTPA and b) 2 mM CDTPA.*

The Figure S7 (a) illustrates the reduction localized in region A of BTPA, highlighting the presence of two irreversible reduction peaks. A possible reduction mechanism is presented below. The presence of two peaks close together in region A is attributed to reactions known as "father-son" reactions, representing the reaction of an electrogenerated product with the initial compound^2^. The reaction is a self-protonation, where basic species generated from the reduction of the CTA are rapidly protonated by the carboxylic group of the unreduced CTA molecule. In this case, a fraction of the CTA is reduced at the first observed peak, while the rest acts as an acid for the protonation of the reduction product. The conjugate base of the CTA (i.e., its carboxylate form), formed following the self-protonation reaction, is reduced at a more negative potential than the neutral CTA. Therefore, the second reduction peak in Figure S7 (a) is attributed to the reduction of the deprotonated CTA. The effect of progressively adding OH^-^ confirms this interpretation. Indeed, by deprotonating the CTA through the addition of a strong base (*n*-Bu_4_NOH), a decrease in the first peak is observed, while the second peak increases. When all the CTA is deprotonated by adding 1 equivalent of the base, the first peak completely disappears, while the second one almost doubles.

Figure S7 (b) illustrates the reduction of CDTPA, which includes both region A and region B in the potential window. In this case, only one reduction peak is present in region A because the carboxylic group is too far from the trithiocarbonyl central unit (Z(CS)S) to influence the voltammetric response. However, by deprotonating the carbonyl function of the CTA, the disappearance of peak n.1 and the formation of peak n.2 at a more negative potential of about 140 mV are observed; the new peak can be attributed to the reduction of the deprotonated CTA.

A possible mechanism of electroreduction of acidic CTAs is as follows:

|  | *eq. S1* |
| --- | --- |
|  | *eq. S2* |
|  | *eq. S3* |
|  | *eq. S4*  *eq. S5* |
|  | *eq. S6* |
|  | *eq. S7* |
|  | *eq. S8* |

**Figure S8.** (a) Kinetic plots and (b) chronoamperometry for only electrochemical RAFT at E_app_ = E_1/2_ + 0.06 V and combined electro-photochemical RAFT of BA with BTPA and ZnTPP. Polymerization conditions: 50% (v/v) BA in DMSO + 0.1 M n-Bu_4_NBF_4_, V_tot_ = 10 mL, [M]:[CTA]:[MED] = 100:1:0.02 (M = BA, CTA = BTPA and MED = ZnTPP) at T = 50 °C, DP_target_= 100. Reaction time 6 h.

*Comparison between polymerization under led irradiation and hood light irradiation.* The purpose of this comparison is to evaluate differences in kinetics, dispersity, and control of polymerizations conducted under hood irradiation vs. LED light placed at 10 cm from the cell (0.25 mW cm^-2^).

**Table S3.** Photo-electrochemical polymerization of BzMA with CDTPA/ZnPC with different types of light sources.

| *Entry^a^* | *Light^b^* | *E_app_ – E_1/2_ (mV)* | *% Conv* ***^c^*** | *k_p,app_ (h^-1^)^d^* | *M_n,GPC_^e^* | *M_n,th_^f^* | *I_eff_^g^* | *Đ****^e^*** |
| --- | --- | --- | --- | --- | --- | --- | --- | --- |
| 1 | Hood | +60 | 60 | 0.24 | 11000 | 10888 | 0.99 | 1.20 |
| 2 | LED | +60 | 61 | 0.24 | 10600 | 11099 | 1.04 | 1.17 |

^a^General polymerization conditions: *T* = 50 °C, 50% (v/v) M in DMSO, *V*_tot_ = 10 mL, reaction time 6 h, [BzMA]:[CDTPA]:[ZnPC] = 100:1:0.02 with [BzMA] = 2.95 M. ^b^Types of source: Hood = hood light, LED = a strip of 6 white light LEDs, with 0.25 mW/cm^2^ irradiance at the reactor center. ^c^Determined by NMR. ^d^The slope of the ln([M]_0_/[M]) vs time plot. ^e^Measured via GPC with DMF eluent at 60 °C. ^f^ *M*_n,th_ = *M*_w,CTA_ + *M*_w,M_ × *DP*_target_ × % conv. ^g^*I*_eff_ = *M*_n,th_/*M*_n,GPC_.

**Figure S9.** Photo-electrochemical polymerization of 50% (v/v) M in DMSO at 50 °C with the ZnPC/CDTPA catalytic system. Complete reaction conditions are described in **Table S3**.

These results indicate that the LED construction faithfully reproduces the polymerization conducted under ambient light. The conversions achieved after 6 hours of reaction are nearly identical, as are the trends of molecular weight and dispersity versus conversion.

**Figure S10.** Chronoamperometry recorded during the reaction in Table 3, entry 2 of the main text.

**Figure S11.** Kinetic plots and trends of MW and dispersity of RAFT polymerizations of BzMA 50% (v/v) in DMSO using the reverse catalytic system ZnTPP/CDTPA (dark brown) and ZnPC/CDTPA (yellow). The solid line in the right graph indicates the theoretical M_n_.

**Figure S12.** (a-b) Kinetic plots and trends of MW and dispersity of RAFT polymerizations of DMAEMA 50% (v/v) in DMSO using the catalytic system ZnPC/CDTPA under 0.25 mW irradiation. (c-d)Kinetic plots and trends of MW and dispersity of RAFT polymerizations of HEMA 50% (v/v) in DMSO using the catalytic system ZnPC/CDTPA under 0.25 mW irradiation. The dashed line in the right graphs indicates the theoretical M_n_.

**Table S4.** PET-RAFT polymerizations of DMAEMA and HEMA with CTA of 33% deprotonation under 0.25 mW/cm^2^ white light.

| Entry^a^ | M/CTA | % CTA  deprotonated^b^ | % Conv | *k*_p,app_ (h^-1^)^d^ | *M*_n,GPC_^e^ | *M*_n,th_^f^ | *I*_eff_^g^ | *Đ****^e^*** |
| --- | --- | --- | --- | --- | --- | --- | --- | --- |
| 1 | DMAEMA/CDTPA^h^ | 33 | 56^c1^ | 0.13 | 12667 | 7415 | 0.58 | 1.22 |
| 2 | HEMA/CDTPA^h^ | 33 | 65^c2^ | 0.18 | 15949 | 8862 | 0.55 | 1.16 |

^a^General polymerization conditions: *T* = 50 °C, 50% (v/v) M in DMSO, *V*_tot_ = 10 mL. ^b^Via addition of *n-*Bu_4_NOH. ^c1^Reaction time = 6 h, ^c2^Reaction time = 5 h. ^d^The slope of the ln([M]_0_/[M]) vs time plot. ^e^Measured via GPC with DMF eluent at 60 °C. ^f^*M*_n,th_ = *M*_w,CTA_ + ([M]/[CTA])*M*_w,M_ × % conv. ^g^*I*_eff_ = *M*_n,th_/*M*_n,GPC_. ^h^[M]:[BTPA]:[ZnTPP] = 100:1:0.02.

# Characterization of the electrolysis products

Electrolysis of the CTA-catalyst systems (BTPA-ZnTPP and CDTPA-ZnPC) in the absence of monomer was conducted under the same operating conditions used for polymerization (see *electrolysis* section), employing 10 mL of DMSO with 0.1 M *n*-Bu_4_NBF_4_ at 50 °C, and with 1 mM catalyst and 20 mM CTA. After each addition during cell preparation and at the conclusion of the electrolysis, a small aliquot was taken for NMR and UV-Vis analysis. A constant potential equal to *E*_1/2_ + 30 mV was applied. The duration of the electrolysis was 4 hours. A clear shift in both the NMR and UV-Vis spectra demonstrated the formation of deprotonated CTA, which formed a complex with the catalyst ZnTPP (Figure S13 and Figure S14) and ZnPC (Figure S15). Indeed, these spectral shifts were comparable to those observed for CTA/PC system after deprotonation by *n*-Bu_4_NOH addition. These control experiments highlight that during electrolysis, the CTA is deprotonated, leading to the formation of a complex between the PC and the CTA.


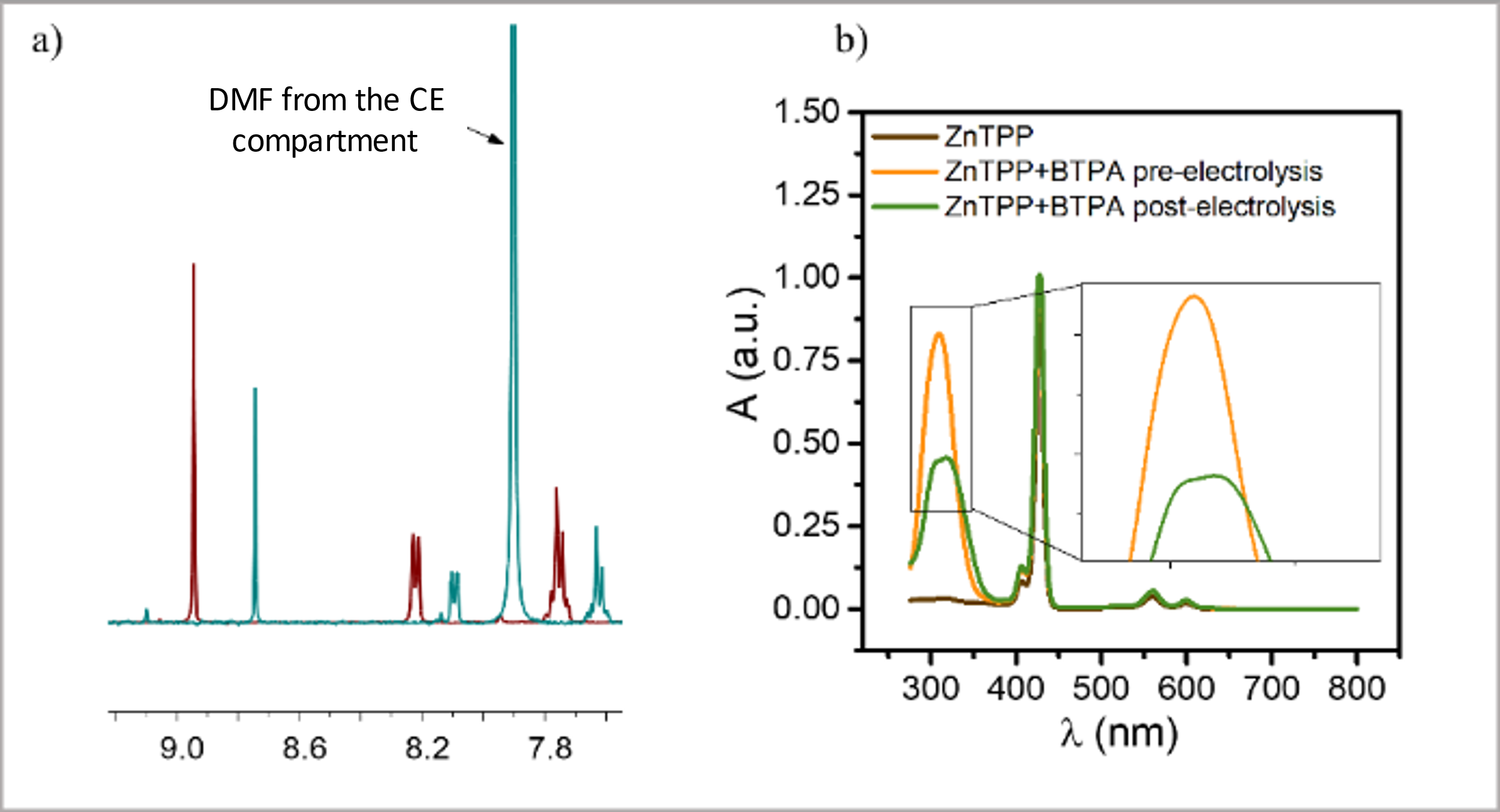


**Figure S13.** a) ^1^H NMR of ZnTPP + BTPA (1:20) in CDCl_3_, recorded before (red) and after electrolysis (blue). The shift in NMR spectra is comparable to that observed following deprotonation in Figure 5b in the main text. b) UV-Vis in DMSO of 0.01 mM ZnTPP with of 0.2 mM BTPA before (orange) and after electrolysis (green). The shift in the UV-Vis spectrum is comparable to the shift observed in **Figure S14** following deprotonation.

**Figure S14.** UV-Vis spectra of 0.01 mM ZnTPP in CDCl_3_ in the absence and presence of 0.2 mM BTPA or 0.2mM BTPA + 0.2 mM n-Bu_4_NOH. It should be noted that these are highly diluted conditions, therefore the spectral shift related to the weak ZnTPP-CTA complex is not visible.


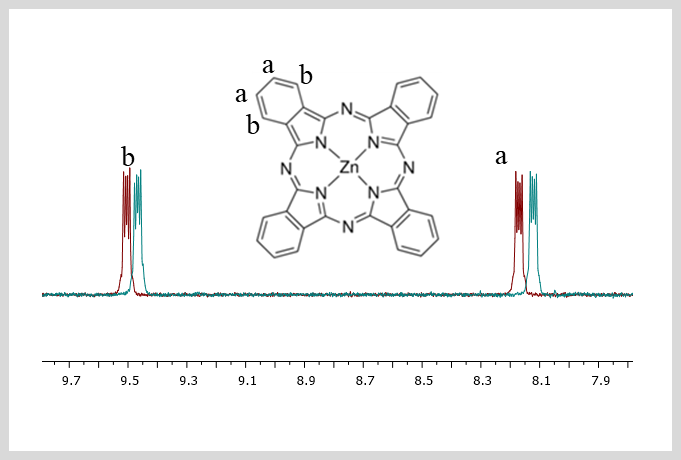


**Figure S15.** ^1^H NMR of 1 mM ZnPC + 20 mM CDTPA recorded in CDCl_3_ before (red) and after electrolysis (blue).

# Additional spectroscopic data

Figure S16 shows shifts of the NMR signal of the α proton to the carboxylic acid group of BTPA in different reaction conditions.


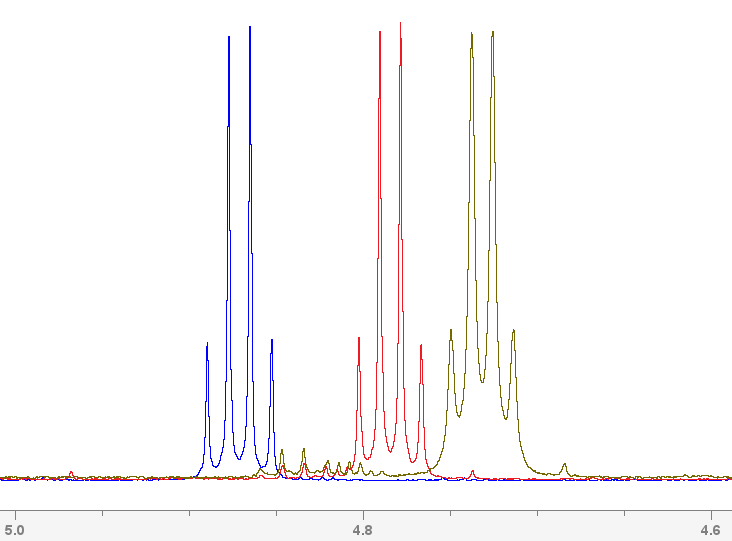


**Figure S16.** 600 MHz ^1^H NMR spectrum of 10 mM BTPA (blue), 10 mM BTPA + 10 mM n-Bu_4_NOH (red), 10 mM BTPA + 10 mM n-Bu_4_NOH + 10 mM ZnTPP (yellow) in CDCl_3_. Close up of the NMR region involving the signal of the α proton to the carboxylic acid groups.

Full NMR spectra are reported in Figure S17 and subsequent figures, where minor shifts were observed in the other peaks further form the carboxylic acid group.


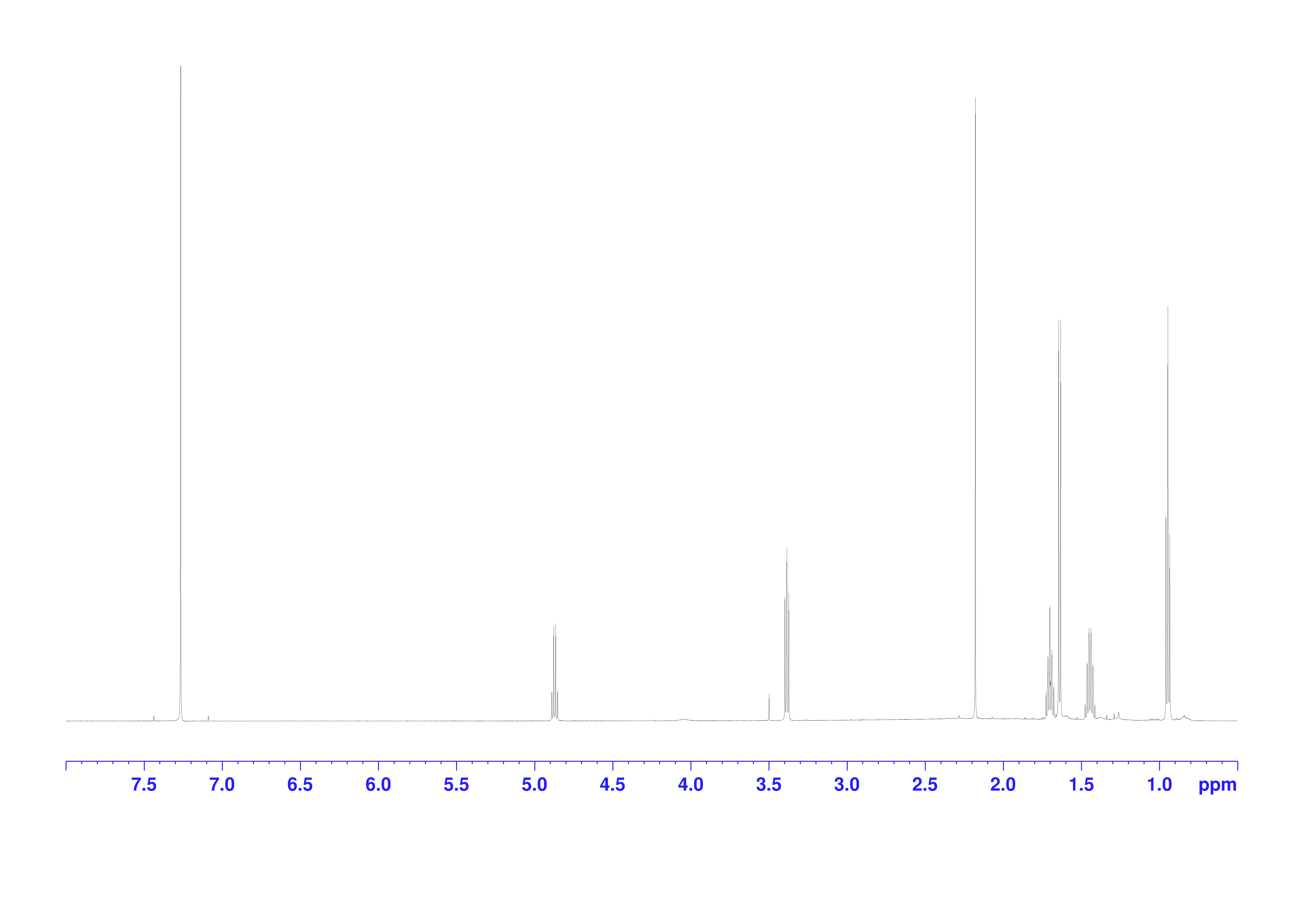


**Figure S17.** 600 MHz ^1^H NMR of 10 mM BTPA in CDCl_3_


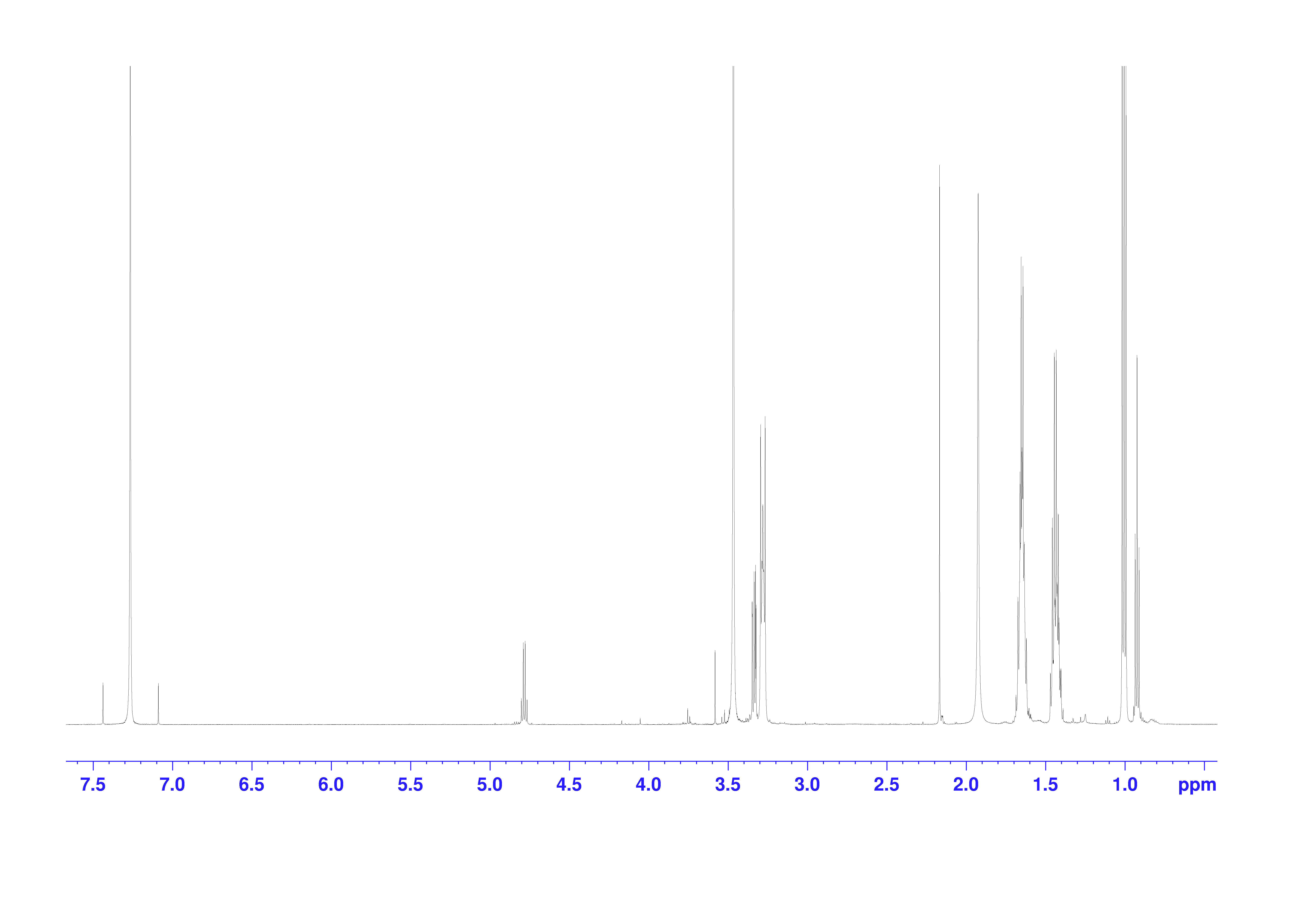


**Figure S18.** 600 MHz ^1^H NMR of 10 mM BTPA + 10 mM n-Bu_4_NOH in CDCl_3_.


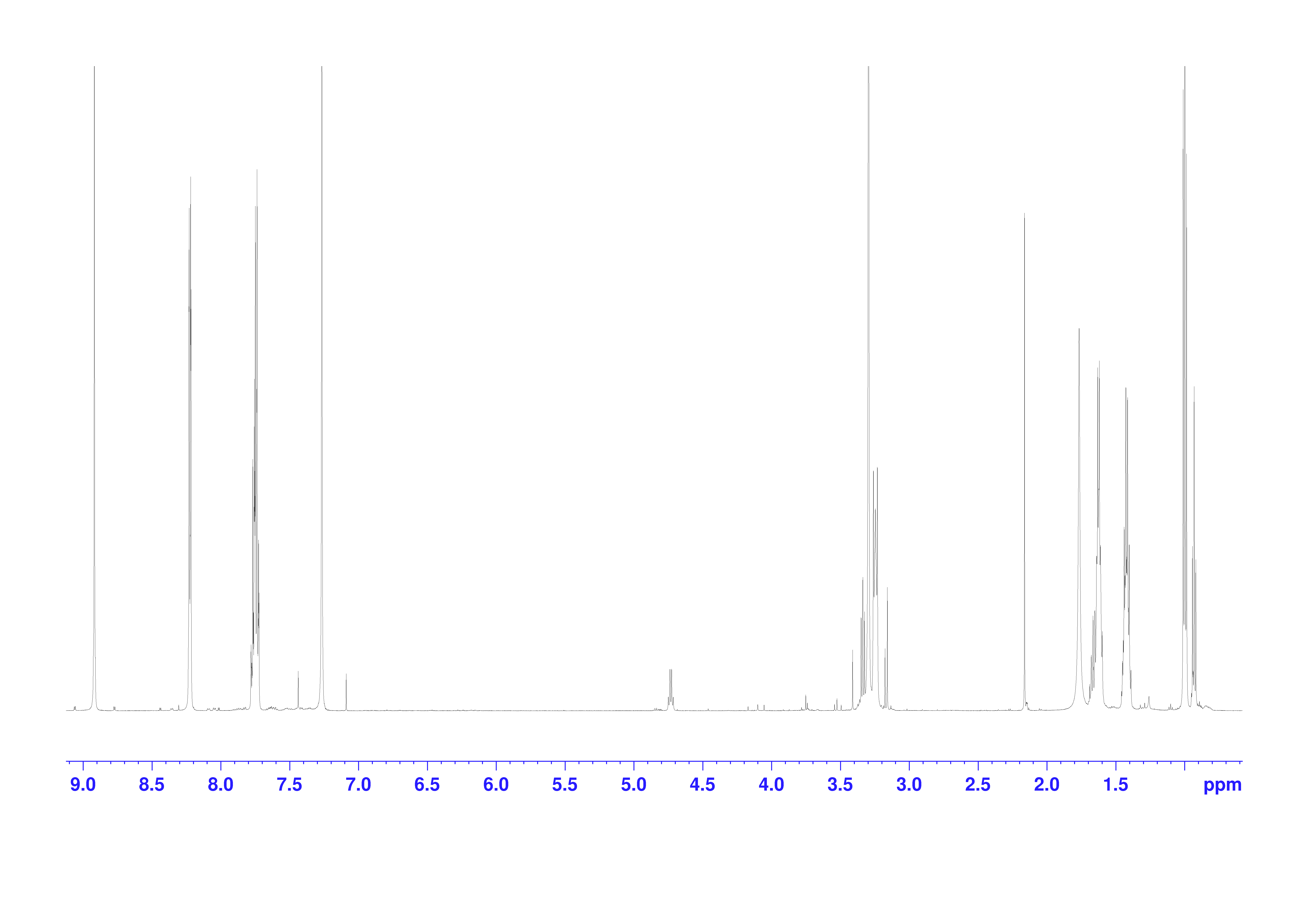


**Figure S19.** 600 MHz ^1^H NMR of 10 mM BTPA + 10 mM n-Bu_4_NOH + 10 mM ZnTPP in CDCl_3_.

**Figure S20.** Full emission spectra (to be compared to Figure 5f in the main text) of 0.01 mM ZnTPP + 20m BTPA + 19 mM n-Bu_4_NOH in toluene. Excitation 400 nm in quartz cuvette 1mm.

**Figure S21.** (a) UV-Vis spectra in toluene of 0.01 mM ZnTPP, 20 mM HAc + 19 mM n-Bu_4_NOH, and 20 mM HAc + 19 mM n-Bu_4_NOH + 0.01 mM ZnTPP. (b) and (c) are spectral enlargements.

**Figure S22.** ^1^H NMR of 1 mM ZnTPP in CDCl_3_ recorded in the absence (red) and presence of 20 mM HAc + 19 mM n-Bu_4_NOH (blue).

**Figure S23.** UV-Vis spectra of 0.01 mM ZnTPP in toluene recorded in the absence and presence of acetic acid (HAc), BTPA, or CTDPE, each at a concentration of 20 mM.

**Figure S 24**. (a)Emission spectra of 0.01 mM ZnTPP+ various concentrations of BTPA, previously deprotonated with n-Bu_4_NOH (BTPACOO^-^) were added in toluene. BTPACOOH indicates the neat CTA. Excitation at 549 nm in quartz cuvette 10mm. (b) Lifetimes were measured by time correlated single photon counting.

**Figure S 25**. (a) UV-Vis spectra in toluene in quartz cuvette 10 mm of 0.01 mM ZnTPP + various concentrations of BTPA, previously deprotonated with n-Bu_4_NOH (BTPACOO^-^) were added in toluene. BTPACOOH indicates the neat CTA. (b) and (c) are spectral enlargements.

# Thermodynamic aspects of photoinduced electron transfer

The photoreduction of the chain transfer agent was studied at 25 °C

(S9)

Eq. (S9) is expected to be the first step in the photoreactive sequence that results in the formation of propagating radicals. of reaction (S9) was calculated from the standard potentials of the donor and acceptor redox couples in the ground state,  and , and the energy of the excited reactant, $E_{00}$, using the Weller equation:^22^

(S10)

The value of was measured as the reversible wave of ZnTPP (Figure S 26) as +0.35 V vs Fc^+^/Fc. The value of cannot be accurately measured by CV since its CV is irreversible. The value was estimated from the value of reduction peak potential recorded on GC electrode at 0.1 V s^-1^ as = -1.91 V vs Fc^+^/Fc. The value of *E*_00_ was estimated from the overlap of the absorption and emission spectrum of ZnTPP (λ = 598 nm), which resulted in *E*_00_ = *hc*/*λ* = -2.07 eV (here *h* is the Planck constant, and *c* is the speed of light).

The radii of the reactants were estimated from their diffusion coefficients via the Stokes-Einstein equation (see Figure S27).

The Gibbs free energy of the reaction is estimated to be slightly positive, suggesting that that the PET is not very efficient.

The Weller equation was also be used to determine the standard reduction potential of the photocatalyst = -1.72 V vs Fc^+^/Fc.

Figure S 26. CV of ZnTPP on a GC electrode in DMSO + 0.1 M n-Bu_4_NBF_4_ at room temperature.

***Kinetic aspects of photoinduced electron transfer***

The reaction (S9) was assumed to proceed via an outer sphere electron transfer (OSET) from a donor (the excited-state catalyst, Cat*) to an acceptor (RSSZ), which subsequently evolves to products (i.e., it cleaves to form propagating radicals and sulfur-based anion).

According to the Marcus theory for electron transfer,^23^ the rate constant of reaction (S9) can be calculated by the following relations:

| $k_{\text{PET}}=Z \text{exp}\left(-\frac{\Delta G^{\ddagger}}{RT}\right)$ | (S11) |
| --- | --- |
|  | (S12) |
| $\Delta G_{0}^{\ddagger}=\frac{\lambda_{\text{i}}+\lambda_{\text{o}}}{4}$ | (S13) |

where is the reaction free energy, $\Delta G_{0}^{\ddagger}$ is the intrinsic barrier (*i.e.* the activation free energy when = 0) of the reaction, λ_o_ is the solvent reorganization energy and λ_i_ is the internal reorganization energy.

The value of λ_o_ can be obtained from the following relation that has been developed for electron transfer in DMF. Since DMSO is a solvent with similar polarity to DMF, the same relation was assumed to hold.

$$\lambda_{\text{o}}=95\left(\frac{1}{2r_{\text{Cat}}}+\frac{1}{2r_{\text{RSSZ}}}-\frac{1}{r_{\text{Cat}}+r_{\text{RSSZ}}}\text{ }\right)\text{ kcal mol}^{-1}=14.6\text{ kcal mol}^{-1}=61.1\text{ kJ mol}^{-1}$$

The value of λ_i_ is unknown but it is generally small, so it was estimated to be 30% of the value of λ_o_. Therefore λ_i_ = 18.3 kJ mol^-1^ was estimated. From these values, $\Delta G_{0}^{\ddagger}$= 19.9 kJ mol^-1^ was obtained. Thus, $\Delta G^{\ddagger}=27.2\text{ kJ mol}^{-1}$ was calculated from eq. (S12).

The frequency factor can be estimated from collision theory:

| $Z=N_{\text{A}}\sqrt{\frac{8\text{π}RT}{\mu}}\left(r_{\text{Cat}}+r_{\text{RSSZ}}\right)^{2}$ | (S14) |
| --- | --- |
|  |  |

where μ is the reduced mass of the reactants. In the case Cat = ZnTPP* (*M* = 678.11 g mol^-1^) and RSSZ = BTPA (*M* = 238.39 g mol^-1^), *μ* = 176.38 g mol^-1^ and Z = 1.03×10^11^ M^-1^ s^-1^.

Thus, from equation (S11), *k*_PET_ = 1.7×10^6^ M^-1^ s^-1^ can be estimated.

***Determination of the diffusion coefficients***

The diffusion coefficient of the photocatalyst was determined via the Randles-Sevcik equation, by recording the CV of the photocatalyst at different scan rates (Figure S27). The diffusion coefficient of the chain transfer agents was calculated from a linear correlation developed in the literature.^24^ For both ZnTPP and ZnPC, *D* = 2.3 × 10^-6^ cm^2^ s^-1^ was obtained.


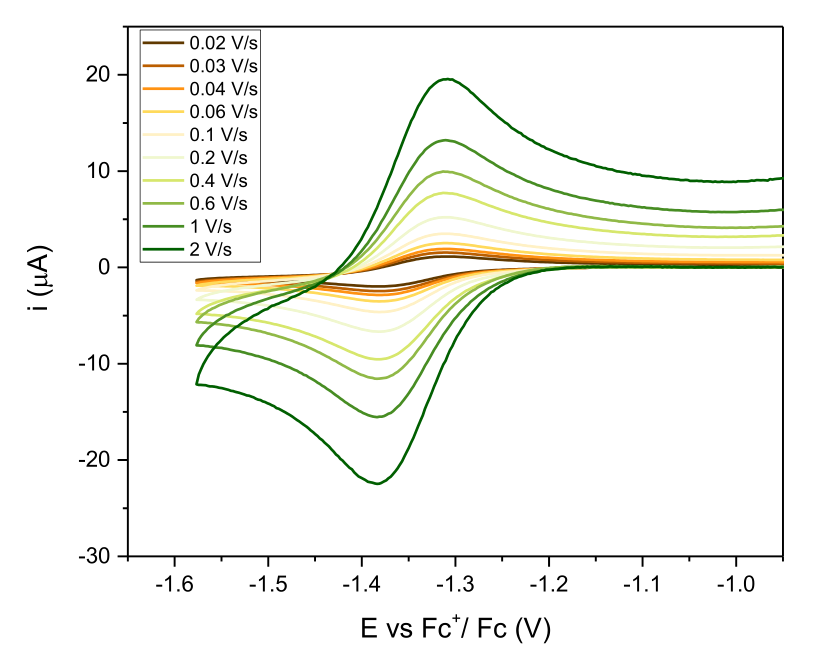


Figure S27. Cyclic voltammetry of ZnPC 0.5 mM in DMSO/MMA (1/1, v/v) + n-Bu₄NBF₄ 0.1 M, recorded at T = 25 °C on a Pt electrode at different potential scan rates.

Figure S28. Molecular weights and dispersity during the polymerization of 50 vol% BA in DMSO with BTPA (blue dots) or PATPA (yellow dots) at 33% deprotonation. [M]:[CTA]:[ZnTPP] = 100:1:0.02 at 50 °C under 0.25 mW cm^-2^ irradiation.

# Computational methods

All Density Functional Theory (DFT) calculations were carried out with the Amsterdam Density Functional (ADF) program 2019.307.^3-4^ To include relativistic effects, the zeroth-order regular approximation (ZORA) was employed.^5^ In the chain transfer reactions, OLYP^6-7^ functional was used together with the TZ2P basis set for the geometry optimizations; furthermore, small frozen core approximation was employed.^8-12^ Frequency calculations were performed for all fully optimized structures to assess whether or not a true minimum was reached and to extract thermodynamic corrections. All minima have real frequencies, while transition states have one imaginary frequency associated with the normal mode connecting reactants to products. M06-2X (a meta-hybrid functional with 54% Hartree-Fock exchange)^13^ was selected for single point calculations of all previously optimized stationary points; in these calculations, all electron TZ2P basis set was used. Moreover, solvation effects (DMSO) have been included by means of COSMO,^14^ conductor-like screening model (level of theory: COSMO-ZORA-M06-2X/TZ2P//ZORA-OLYP/TZ2P). For the precomplexation of ZnTTP with BTPA, BP86^15-16^ functional was used with TZ2P basis set and the small frozen core approximation for the geometry optimizations.^17^ Then, single point calculations were conducted using B3LYP^7,15,18^ hybrid functional with the Grimme D3 dispersion correction and the Becke–Johnson damping function.^19-21^ Moreover, the implicit solvation effects were introduced using toluene (level of theory: COSMO-ZORA-B3LYP-D3(BJ)/TZ2P//ZORA-BP86/TZ2P).


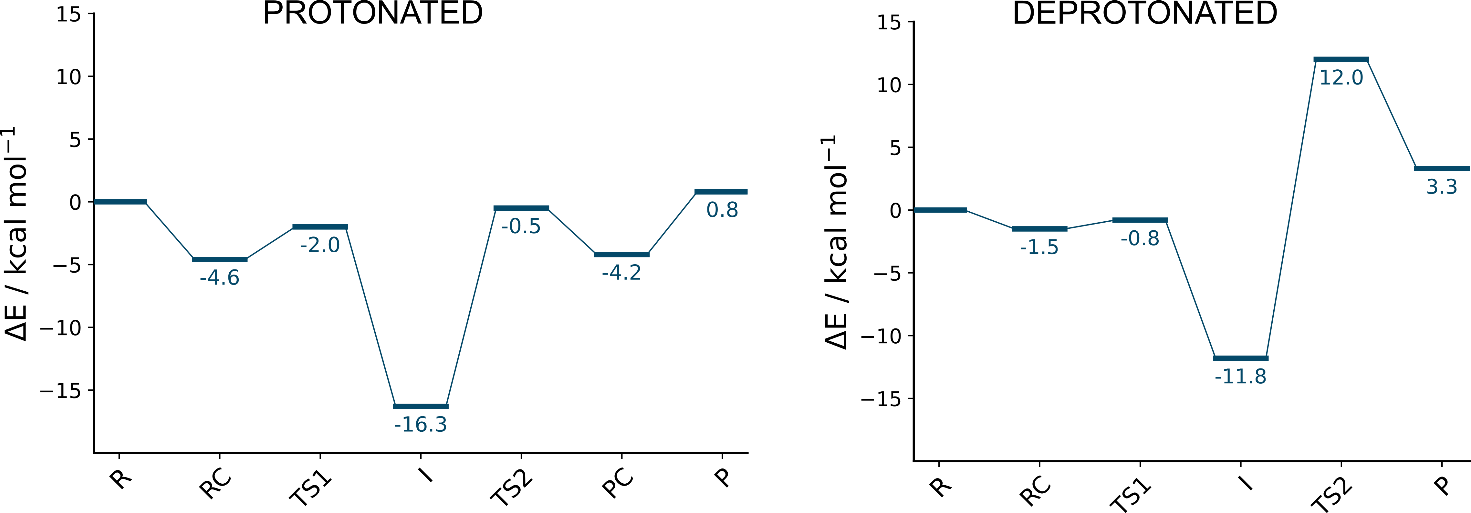


***Figure S29.*** *Energy profile of the chain transfer reaction for protonated and deprotonated BTPA in DMSO. Electronic energies are reported in kcal mol^-1^ with respect to the free reagents. Level of theory: COSMO-ZORA-M06-2X/TZ2P//ZORA-OLYP/TZ2P. R = reagent, RC = reagent complex, PC = product complex, P = product.*

Electronic energies (Figure S26) do not show any significant difference; due to the absence of entropic effects, the intermediate resulted to be stabilized in energy. Nevertheless, the deprotonation of BTPA still destabilized the trithiocarbonate radical and the following transition state, resulting in a higher energy barrier.


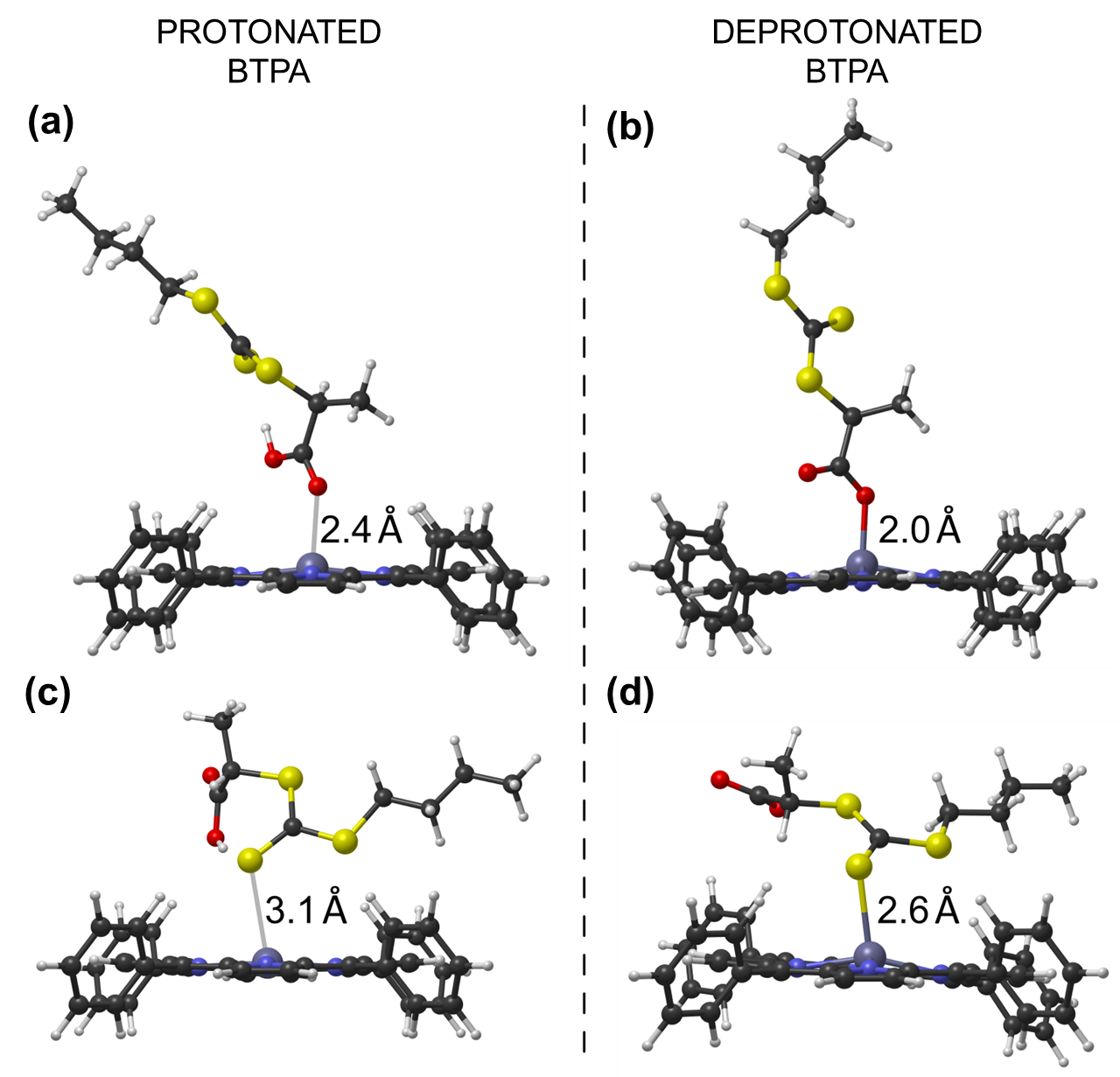


***Figure S30.*** Fully optimized structures of the ZnTPP in the presence of a, c) protonated and b, d) deprotonated BTPA. a, b) coordination via the carboxylate group. c, d) coordination via the sulfur group. Level of theory: COSMO-ZORA-M06-2X/TZ2P//ZORA-OLYP/TZ2P.

**Table S5.** Coordinates (Å) and energies (E, Hartree) of all the studied geometries. Level of theory: ZORA-BP86/TZ2P.

**ZnTTP**  E= -19.26730782

C -0.832142 -3.923930 -7.629076

C -1.281710 -0.568760 -4.089270

C -1.119476 -4.111599 -0.711039

C -0.670337 -7.466721 -4.250868

N -1.139017 -2.610898 -2.686958

N -1.068167 -2.536563 -5.585493

N -0.762464 -5.420221 -5.650970

N -0.931735 -5.503091 -2.756787

C -0.697089 -6.787613 -5.483858

C -0.770105 -5.186941 -7.010202

C -0.947930 -2.696078 -6.950069

C -1.150963 -1.180089 -5.350761

C -0.757451 -6.851559 -2.987497

C -0.960455 -5.335620 -1.388182

C -1.224215 -2.852330 -1.331803

C -1.297749 -1.251690 -2.858172

C -0.681688 -7.432007 -6.776317

C -0.726605 -6.445851 -7.716698

C -0.936485 -1.400742 -7.588685

C -1.061482 -0.466738 -6.603374

C -1.507139 -0.624336 -1.574240

C -1.461706 -1.610444 -0.633837

C -0.655128 -7.548171 -1.726478

C -0.780234 -6.614187 -0.741150

H -0.657852 -8.502900 -6.941265

H -0.746324 -6.559582 -8.794314

H -0.828346 -1.228962 -8.653296

H -1.074570 0.611529 -6.711696

H -1.684230 0.433105 -1.415988

H -1.594660 -1.510088 0.437078

H -0.490601 -8.613219 -1.611562

H -0.737167 -6.772722 0.330116

C -0.770497 -3.883410 -9.122688

C 0.409439 -4.226022 -9.801459

C -1.891287 -3.501815 -9.876746

H 1.288006 -4.517162 -9.225916

H -2.815000 -3.240584 -9.360591

C 0.467900 -4.185349 -11.195162

C -1.834438 -3.466077 -11.270648

H 1.395121 -4.447967 -11.704583

H -2.717106 -3.174050 -11.839716

C -0.654273 -3.806481 -11.934384

H -0.609271 -3.776673 -13.022890

C -1.413093 0.920393 -4.053989

C -2.564473 1.551324 -4.550688

C -0.387381 1.718229 -3.523128

H -3.370406 0.941472 -4.958679

H 0.514269 1.239601 -3.141269

C -2.687830 2.940928 -4.514926

C -0.508548 3.108099 -3.491908

H -3.592463 3.412706 -4.898706

H 0.301487 3.711665 -3.082301

C -1.659748 3.723961 -3.986588

H -1.755292 4.809290 -3.960464

C -1.182653 -4.152072 0.782521

C -2.281957 -4.727838 1.438563

C -0.144233 -3.614677 1.559215

H -3.097388 -5.141800 0.845586

H 0.717571 -3.171396 1.060517

C -2.342490 -4.762803 2.832331

C -0.202312 -3.654224 2.952967

H -3.207609 -5.207586 3.324234

H 0.616887 -3.238204 3.539545

C -1.302263 -4.227211 3.594093

H -1.348595 -4.256206 4.682566

C -0.540730 -8.956094 -4.285952

C 0.649540 -9.562954 -4.716471

C -1.607219 -9.778133 -3.889357

H 1.486744 -8.934533 -5.019881

H -2.538632 -9.317933 -3.559853

C 0.770697 -10.952870 -4.747458

C -1.487894 -11.168067 -3.925169

H 1.705265 -11.406138 -5.078130

H -2.329375 -11.790269 -3.620237

C -0.298075 -11.760018 -4.353140

H -0.204165 -12.845489 -4.379255

Zn -0.974705 -4.017534 -4.170033

**BTPA**  E= -5.40592224

H 3.318458 -4.846698 -6.778757

S 2.668310 -6.015515 -2.680237

C 3.098419 -5.053650 -4.065208

H 5.912542 -1.638959 -6.178343

S 4.771610 -4.541808 -4.155253

S 1.891526 -4.721706 -5.164770

O 4.120190 -4.939098 -7.373688

O 6.212081 -4.150116 -7.450129

H 5.627484 -1.770367 -4.421327

H 3.854178 -7.396002 2.415637

H 6.878193 -2.767564 -5.224808

C 5.878405 -2.331753 -5.328581

C 4.831706 -3.405433 -5.606616

H 3.815560 -2.985013 -5.671431

C 5.137134 -4.197372 -6.895463

C 4.161861 -6.122260 -1.615530

H 4.965236 -6.612190 -2.181507

H 4.481717 -5.104479 -1.355264

C 3.803342 -6.927438 -0.363972

H 2.971639 -6.436437 0.164534

H 3.443611 -7.925630 -0.657830

C 5.000144 -7.073630 0.585261

H 5.830537 -7.558997 0.049123

H 5.360834 -6.072992 0.870545

C 4.659453 -7.876548 1.842895

H 4.326870 -8.892325 1.588051

H 5.531054 -7.966721 2.503582

**ZnTTP-BTPA(κ-O)** E= -24.67705158

H 2.641153 -2.117124 -1.883771

S 6.810824 -3.569552 -1.617701

C 5.299258 -2.791044 -2.016865

H 2.785990 -1.504157 -6.030886

S 4.724188 -3.474906 -3.531150

S 4.552177 -1.616568 -1.109987

O 1.822605 -2.490074 -2.337182

O 1.224381 -3.130826 -4.393191

H 4.531667 -1.879049 -5.979114

H 9.746500 -5.234742 2.627317

H 3.317232 -3.185624 -6.107120

C 3.537728 -2.210774 -5.658859

C 3.459388 -2.270153 -4.137381

H 3.737682 -1.311720 -3.673009

C 2.063985 -2.675525 -3.634988

C 7.108120 -3.073262 0.116914

H 7.243218 -1.984862 0.136338

H 6.205141 -3.317877 0.691459

C 8.341154 -3.809042 0.642885

H 8.181434 -4.896207 0.578103

H 9.211691 -3.580356 0.008510

C 8.652774 -3.421714 2.095054

H 8.803958 -2.332665 2.155065

H 7.778086 -3.646166 2.725060

C 9.884134 -4.144732 2.645499

H 10.780050 -3.911831 2.053367

H 10.084612 -3.851051 3.683707

C -1.038443 -4.134122 -7.724494

C -2.197261 -0.510952 -4.639330

C -1.534798 -3.536079 -0.844028

C -0.323588 -7.142826 -3.926504

N -1.724577 -2.298338 -2.985261

N -1.564993 -2.563901 -5.878228

N -0.736880 -5.339157 -5.577809

N -0.976627 -5.095897 -2.688665

C -0.445099 -6.639508 -5.238033

C -0.770409 -5.282613 -6.951745

C -1.384303 -2.868406 -7.207554

C -1.904584 -1.232199 -5.815535

C -0.558755 -6.405567 -2.748035

C -1.108237 -4.778063 -1.356765

C -1.848546 -2.401122 -1.619059

C -2.144847 -1.034768 -3.331147

C -0.309240 -7.434640 -6.439046

C -0.511161 -6.597837 -7.496264

C -1.591669 -1.681448 -8.007998

C -1.911369 -0.671566 -7.149098

C -2.564143 -0.322839 -2.143675

C -2.382732 -1.166203 -1.088014

C -0.392691 -6.921025 -1.406767

C -0.732953 -5.917476 -0.548641

H -0.109912 -8.499715 -6.467718

H -0.508132 -6.851882 -8.549984

H -1.492685 -1.625077 -9.085931

H -2.121835 0.362855 -7.395171

H -2.969875 0.682099 -2.124530

H -2.612029 -0.978356 -0.045436

H -0.049707 -7.917907 -1.154855

H -0.718903 -5.941750 0.534885

C -0.959358 -4.271696 -9.210929

C 0.266747 -4.526803 -9.845947

C -2.109859 -4.150308 -10.006895

H 1.167752 -4.618858 -9.239796

H -3.069320 -3.961775 -9.525648

C 0.341632 -4.652545 -11.233762

C -2.036658 -4.280222 -11.394373

H 1.304486 -4.844166 -11.707857

H -2.942868 -4.189749 -11.993576

C -0.810293 -4.530295 -12.013223

H -0.752786 -4.630390 -13.097012

C -2.612230 0.916896 -4.790990

C -3.818438 1.253355 -5.426590

C -1.806192 1.955829 -4.298112

H -4.458373 0.456181 -5.804313

H -0.865830 1.708330 -3.805803

C -4.206319 2.586612 -5.565045

C -2.191277 3.289595 -4.439877

H -5.150250 2.826039 -6.055070

H -1.547967 4.081537 -4.055734

C -3.393382 3.609966 -5.073710

H -3.695729 4.651398 -5.182951

C -1.677574 -3.416015 0.639056

C -2.656599 -4.148337 1.329744

C -0.837113 -2.565747 1.375424

H -3.321523 -4.804568 0.768503

H -0.070234 -1.996792 0.850535

C -2.791626 -4.033590 2.713939

C -0.968956 -2.453516 2.760156

H -3.563078 -4.605135 3.230345

H -0.302682 -1.792113 3.314518

C -1.947329 -3.186643 3.434615

H -2.052218 -3.097480 4.515843

C 0.070335 -8.577079 -3.775269

C 1.351524 -9.013088 -4.149288

C -0.831481 -9.521197 -3.258555

H 2.062115 -8.288712 -4.546992

H -1.832203 -9.197148 -2.973407

C 1.721499 -10.351225 -4.007688

C -0.463669 -10.860255 -3.120451

H 2.723265 -10.668505 -4.298237

H -1.181023 -11.579030 -2.723917

C 0.814778 -11.280003 -3.493190

H 1.102479 -12.325641 -3.384387

Zn -1.061011 -3.770361 -4.272355

**ZnTTP-BTPA(κ-S)** E= -24.67362635

H 2.816073 -4.087683 -6.448620

S 2.839211 -5.268870 -2.307887

C 3.305921 -4.496469 -3.789611

H 6.765062 -2.775439 -6.815387

S 4.889197 -4.909570 -4.417797

S 2.214702 -3.439061 -4.484631

O 3.245771 -4.496233 -7.257950

O 5.313951 -4.880701 -8.015275

H 6.962444 -2.964205 -5.051483

H 4.498903 -7.817418 2.169219

H 7.209204 -4.351234 -6.155359

C 6.610448 -3.453243 -5.966958

C 5.126111 -3.787665 -5.863530

H 4.518984 -2.898975 -5.629788

C 4.587082 -4.446225 -7.149711

C 4.264393 -6.299918 -1.780203

H 4.463773 -7.049025 -2.558142

H 5.147365 -5.655346 -1.674646

C 3.904877 -6.971497 -0.452489

H 3.683694 -6.201080 0.302024

H 2.984319 -7.561707 -0.576258

C 5.034541 -7.877990 0.053488

H 5.254409 -8.642210 -0.708316

H 5.954573 -7.283308 0.167489

C 4.692973 -8.558273 1.381261

H 3.796224 -9.185993 1.286489

H 5.515804 -9.200034 1.721267

C -0.813036 -3.920991 -7.628592

C -1.273638 -0.568236 -4.088327

C -1.097655 -4.109479 -0.708484

C -0.661965 -7.466321 -4.251180

N -1.122553 -2.607621 -2.682577

N -1.037542 -2.530856 -5.586101

N -0.741441 -5.419409 -5.650580

N -0.895299 -5.502736 -2.751769

C -0.696904 -6.786407 -5.484604

C -0.763425 -5.184783 -7.008780

C -0.922447 -2.691192 -6.950267

C -1.132088 -1.176389 -5.351188

C -0.732413 -6.851100 -2.985035

C -0.928845 -5.334531 -1.384580

C -1.215744 -2.850674 -1.330063

C -1.295804 -1.252259 -2.856746

C -0.714519 -7.430942 -6.778403

C -0.753131 -6.443887 -7.717165

C -0.921197 -1.395750 -7.590200

C -1.051032 -0.462103 -6.604878

C -1.528737 -0.626627 -1.574915

C -1.479602 -1.611740 -0.633837

C -0.633868 -7.549165 -1.724205

C -0.754466 -6.614165 -0.737490

H -0.715690 -8.501674 -6.945580

H -0.788183 -6.555587 -8.794490

H -0.819909 -1.224525 -8.655575

H -1.075870 0.615859 -6.714642

H -1.724959 0.427802 -1.418923

H -1.628890 -1.512588 0.435052

H -0.485065 -8.616633 -1.608844

H -0.722480 -6.775723 0.333865

C -0.750418 -3.884096 -9.122365

C 0.428564 -4.240124 -9.796621

C -1.867944 -3.494824 -9.877539

H 1.306117 -4.534760 -9.221206

H -2.789964 -3.224382 -9.362865

C 0.486522 -4.203512 -11.190702

C -1.809910 -3.464449 -11.271517

H 1.412457 -4.475747 -11.697275

H -2.689654 -3.167034 -11.842483

C -0.631661 -3.817535 -11.932354

H -0.585406 -3.791999 -13.021049

C -1.418604 0.919503 -4.051812

C -2.568505 1.541986 -4.562753

C -0.407929 1.725418 -3.504319

H -3.363224 0.926036 -4.983307

H 0.492151 1.253396 -3.110783

C -2.704872 2.930311 -4.525089

C -0.541990 3.114068 -3.470969

H -3.608337 3.394944 -4.920246

H 0.256623 3.723780 -3.048104

C -1.691464 3.721194 -3.980295

H -1.797146 4.805571 -3.952634

C -1.167430 -4.149199 0.784373

C -2.248476 -4.762317 1.437761

C -0.154895 -3.570516 1.566291

H -3.046034 -5.205783 0.841925

H 0.690510 -3.093958 1.070248

C -2.315227 -4.795695 2.831258

C -0.219221 -3.607303 2.959892

H -3.166830 -5.269503 3.319730

H 0.579102 -3.156383 3.549690

C -1.300032 -4.219647 3.597371

H -1.352025 -4.245917 4.685692

C -0.549579 -8.956363 -4.289189

C 0.610055 -9.577268 -4.780730

C -1.602789 -9.768584 -3.838826

H 1.434520 -8.957956 -5.133559

H -2.512799 -9.298912 -3.465914

C 0.715165 -10.968367 -4.816818

C -1.499671 -11.159627 -3.878741

H 1.625163 -11.431310 -5.198904

H -2.331859 -11.772746 -3.532513

C -0.339581 -11.764579 -4.366266

H -0.259109 -12.851021 -4.397712

Zn -0.849664 -4.005350 -4.162603

**BTPA anion** E= -5.33847914

S -0.129350 -1.747376 1.504632

C 1.296876 -1.987826 0.432375

H 3.625826 -4.805910 -2.009395

S 0.701497 -2.646768 -1.062584

S 2.852340 -1.577560 0.854697

O 0.521100 -3.063953 -3.659428

O 2.557279 -3.947824 -4.203909

H 3.252370 -4.264656 -0.340079

H 1.540082 3.453332 2.215883

H 2.092690 -5.284034 -1.246801

C 2.828322 -4.472408 -1.331638

C 2.198539 -3.232730 -1.952976

H 2.910511 -2.395015 -1.932921

C 1.706159 -3.442816 -3.437894

C 0.493514 -0.612701 2.785544

H -0.128231 -0.817646 3.669610

H 1.528338 -0.926090 2.995283

C 0.444036 0.863507 2.392027

H 1.044885 1.000371 1.480725

H -0.589830 1.143894 2.136002

C 0.974846 1.782950 3.498872

H 0.388799 1.625341 4.419800

H 2.009528 1.492356 3.739929

C 0.934803 3.264464 3.113614

H -0.092379 3.589235 2.892788

H 1.322125 3.903481 3.919599

**ZnTTP-BTPAan** E= -24.63705538

S 4.825127 -2.189207 1.486029

C 4.371540 -2.350026 -0.238269

H 2.974169 -4.784795 -3.605844

S 2.771674 -3.044567 -0.275846

S 5.314378 -1.854778 -1.507898

O 0.239923 -3.531367 -1.167344

O 0.573266 -3.720116 -3.415798

H 4.326879 -4.458864 -2.483873

H 10.238565 -2.995881 2.106800

H 2.967771 -5.507453 -1.978217

C 3.243421 -4.615368 -2.555497

C 2.476240 -3.391094 -2.060454

H 2.799100 -2.495286 -2.607933

C 0.935192 -3.557775 -2.196414

C 6.412064 -1.297251 1.427652

H 6.362536 -0.635113 0.550148

H 6.423089 -0.683386 2.339887

C 7.635913 -2.209811 1.349944

H 7.635715 -2.902328 2.206340

H 7.555051 -2.824606 0.441587

C 8.949092 -1.418155 1.321345

H 8.935909 -0.733155 0.459082

H 9.013102 -0.781139 2.219022

C 10.184974 -2.318501 1.242306

H 10.161584 -2.940253 0.336378

H 11.112354 -1.729812 1.220471

C -0.312481 -3.941680 -7.445952

C -2.245930 -0.627206 -4.411286

C -3.150588 -4.156085 -1.149893

C -1.354505 -7.491336 -4.237880

N -2.526511 -2.649082 -3.011993

N -1.351544 -2.563504 -5.671294

N -0.918217 -5.446778 -5.571149

N -2.180791 -5.547201 -2.949600

C -0.923228 -6.807424 -5.395688

C -0.449091 -5.208610 -6.837688

C -0.730375 -2.715823 -6.884649

C -1.607695 -1.224093 -5.520132

C -1.927113 -6.886124 -3.098287

C -2.728492 -5.380474 -1.703499

C -3.091545 -2.902249 -1.789994

C -2.693138 -1.311926 -3.261040

C -0.453692 -7.456432 -6.603872

C -0.158350 -6.467433 -7.495024

C -0.561855 -1.420636 -7.513658

C -1.106286 -0.497972 -6.670096

C -3.419175 -0.698882 -2.164999

C -3.658913 -1.682286 -1.252134

C -2.315248 -7.593029 -1.892791

C -2.808349 -6.660216 -1.029468

H -0.384889 -8.527363 -6.759327

H 0.196147 -6.580537 -8.513554

H -0.076485 -1.238146 -8.465869

H -1.148165 0.576898 -6.806282

H -3.717467 0.342067 -2.109372

H -4.186858 -1.596548 -0.308776

H -2.205613 -8.658049 -1.721603

H -3.178467 -6.820308 -0.022982

C 0.307438 -3.902330 -8.804485

C 1.652591 -4.263430 -8.989568

C -0.433665 -3.508970 -9.931424

H 2.240643 -4.561921 -8.122116

H -1.480720 -3.234995 -9.803304

C 2.237146 -4.230507 -10.256393

C 0.148759 -3.477423 -11.199401

H 3.285245 -4.508018 -10.374298

H -0.448132 -3.174586 -12.060609

C 1.487664 -3.837665 -11.367516

H 1.943887 -3.812168 -12.357583

C -2.466337 0.849805 -4.456738

C -3.342287 1.423389 -5.393310

C -1.802369 1.703408 -3.559928

H -3.869443 0.771135 -6.089433

H -1.114093 1.271432 -2.833905

C -3.548992 2.803180 -5.432277

C -2.006456 3.083463 -3.599128

H -4.238569 3.225706 -6.164173

H -1.474752 3.727095 -2.897455

C -2.881222 3.639703 -4.535113

H -3.041182 4.717921 -4.565407

C -3.679305 -4.177662 0.248500

C -4.939006 -4.715034 0.553419

C -2.901127 -3.651142 1.292225

H -5.549961 -5.122485 -0.252373

H -1.915308 -3.250323 1.056810

C -5.413066 -4.722633 1.867047

C -3.374299 -3.661631 2.605269

H -6.397281 -5.139444 2.084917

H -2.753790 -3.255017 3.404658

C -4.631872 -4.195342 2.897646

H -5.000709 -4.202022 3.923975

C -1.199347 -8.976798 -4.223825

C 0.075350 -9.567468 -4.244870

C -2.320486 -9.823025 -4.190522

H 0.953423 -8.922435 -4.261394

H -3.315375 -9.378172 -4.182163

C 0.224354 -10.954957 -4.231429

C -2.173368 -11.210847 -4.178958

H 1.224280 -11.390298 -4.240455

H -3.058668 -11.847632 -4.158879

C -0.899599 -11.783386 -4.198811

H -0.783674 -12.867567 -4.188298

Zn -1.338266 -3.990646 -4.093961

**Table S6.** Coordinates (Å) and energies (E, Hartree) of all the studied geometries and number of imaginary frequencies (Nimag, cm^-1^) of transition states. Level of theory: ZORA-OLYP/TZ2P.

**PROTONATED**

**BTPA**  E= -5.27742366

H 3.036644 -1.358263 -1.753004

S 0.586292 -1.956320 2.022478

C 1.684034 -2.030778 0.658762

H 3.000265 -5.432706 -2.605892

S 0.965144 -3.138263 -0.496002

S 3.123641 -1.234512 0.505513

O 2.810342 -1.501970 -2.701935

O 1.994397 -3.146477 -3.967621

H 2.226207 -5.726713 -1.032775

H 0.568495 3.593572 1.845440

H 1.247536 -5.332845 -2.467724

C 2.183762 -5.127608 -1.945233

C 2.335676 -3.645031 -1.618541

H 3.250995 -3.462303 -1.045627

C 2.351404 -2.755431 -2.882925

C 1.076732 -0.475402 2.963404

H 0.742387 -0.716300 3.978949

H 2.169003 -0.442732 2.968084

C 0.459295 0.833268 2.469911

H 0.769705 1.011703 1.435179

H -0.632781 0.736917 2.458830

C 0.863828 2.030804 3.340211

H 0.562852 1.844142 4.379696

H 1.957988 2.117325 3.349999

C 0.256246 3.352862 2.867493

H -0.838734 3.318759 2.881200

H 0.569965 4.179656 3.512884

**MArad**  E= -2.62551488

H 3.159698 -1.790505 0.658196

H 5.330374 -3.272810 5.929451

O 2.692609 -2.715949 2.387701

O 2.098668 -3.780001 4.242500

H 4.374569 -1.890851 6.424437

H 3.583199 -3.478959 6.231797

C 4.338370 -2.809334 5.819272

C 4.064514 -2.490582 4.400512

H 4.755536 -1.831392 3.885896

C 2.909471 -3.035515 3.707598

C 3.578283 -1.858079 1.664163

H 3.618583 -0.852249 2.098476

H 4.589601 -2.276275 1.598811

**RC**  E= -7.91356929

H 4.340451 -2.958469 -1.365954

S 1.552552 -0.392579 0.950153

C 1.214786 -0.277685 -0.766817

H 1.252241 -4.684733 0.673550

S 1.367345 -1.738530 -1.718815

S 0.761716 1.066036 -1.607774

O 4.034991 -3.756429 -1.849332

O 2.233589 -5.023753 -2.148210

H 0.273084 -3.206811 0.701341

H -2.493735 3.108848 2.383827

H 0.159143 -4.333615 -0.670602

C 0.836314 -3.881657 0.055114

C 1.968065 -3.141496 -0.650574

H 2.669342 -2.708037 0.066264

C 2.745780 -4.066169 -1.617714

C 1.262115 1.285775 1.587908

H 1.998765 1.385677 2.392942

H 1.528910 1.977787 0.783857

C -0.158160 1.532575 2.098893

H -0.864812 1.374121 1.278047

H -0.400259 0.793962 2.872562

C -0.335632 2.947857 2.664147

H 0.384586 3.109331 3.477570

H -0.086859 3.680675 1.885888

C -1.749911 3.220056 3.180363

H -2.022139 2.530954 3.987617

H -1.834394 4.238932 3.572290

C 5.576255 -0.216261 -2.562730

H 4.913999 -0.896799 -3.089150

C 6.098852 0.966102 -3.281028

H 5.267801 1.621506 -3.585635

H 6.801887 1.549829 -2.687383

H 6.585867 0.660611 -4.218365

C 5.826787 -0.575790 -1.185674

O 5.341142 -1.564761 -0.627099

O 6.659053 0.286253 -0.542077

C 6.950847 -0.024429 0.831334

H 6.036479 -0.025275 1.431141

H 7.432269 -1.002864 0.913767

H 7.626747 0.762586 1.167344

**TS1**  E= -7.90260292

Nimag= -175.693

H 4.318022 -2.977239 -1.203571

S 1.113497 -0.960593 0.947537

C 1.574586 -0.770832 -0.727437

H 1.143551 -5.375937 0.321014

S 1.493176 -2.205797 -1.732206

S 2.041088 0.633425 -1.479990

O 4.256532 -3.871601 -1.606439

O 2.824596 -5.532106 -1.969295

H 0.065878 -3.964571 0.285769

H -0.961914 4.147201 1.561756

H 0.300971 -4.956695 -1.170688

C 0.793337 -4.538772 -0.291035

C 1.976187 -3.663958 -0.686117

H 2.458361 -3.234524 0.195927

C 3.048433 -4.446046 -1.487322

C 1.445748 0.646385 1.736984

H 1.769500 0.372917 2.747423

H 2.294472 1.092598 1.211991

C 0.245384 1.593682 1.779045

H -0.077799 1.808726 0.755472

H -0.594237 1.093340 2.276167

C 0.565763 2.906552 2.505557

H 0.904539 2.685171 3.526551

H 1.408562 3.400387 2.004779

C -0.621094 3.870169 2.565360

H -1.471753 3.426446 3.094406

H -0.350011 4.792495 3.089305

C 4.456435 0.289487 -2.174119

H 4.152781 -0.511192 -2.838560

C 4.792056 1.607786 -2.787949

H 4.095093 1.847633 -3.595850

H 4.789730 2.423191 -2.061150

H 5.799553 1.576896 -3.230767

C 5.028531 -0.191984 -0.930436

O 4.932812 -1.348567 -0.509084

O 5.681518 0.777854 -0.226478

C 6.276779 0.365030 1.013472

H 5.518728 -0.006839 1.709088

H 7.019228 -0.420807 0.847892

H 6.754738 1.259021 1.416486

**I**  E= -7.91415505

H 4.491511 -2.770555 -0.713375

S 0.414568 -0.885351 0.299481

C 1.536518 -0.934215 -1.037735

H 1.578944 -5.551427 0.121080

S 1.558440 -2.395145 -1.980737

S 2.216352 0.525386 -1.680765

O 4.637630 -3.590095 -1.224224

O 3.512603 -5.294679 -2.103681

H 0.277585 -4.360572 -0.101736

H 0.456079 4.439657 1.919292

H 0.888054 -5.301427 -1.482890

C 1.168978 -4.795562 -0.557501

C 2.211265 -3.715612 -0.815349

H 2.445953 -3.174214 0.103413

C 3.495378 -4.282542 -1.442261

C 1.121969 0.251597 1.555190

H 1.007190 -0.290997 2.500549

H 2.190437 0.334525 1.349412

C 0.443873 1.619599 1.630688

H 0.555407 2.130900 0.668775

H -0.633092 1.480108 1.784909

C 1.010845 2.499589 2.752389

H 0.907478 1.977595 3.713067

H 2.089142 2.636676 2.595558

C 0.338365 3.870616 2.848103

H -0.735278 3.776694 3.045166

H 0.772502 4.465484 3.658416

C 4.018916 0.141676 -2.001082

H 4.025373 -0.860421 -2.432398

C 4.553844 1.157821 -3.005791

H 4.009693 1.065668 -3.948719

H 4.460290 2.184712 -2.646850

H 5.613065 0.963744 -3.200849

C 4.736594 0.059265 -0.670201

O 4.794467 -0.957268 0.005402

O 5.275112 1.234456 -0.287265

C 5.956546 1.242303 0.984400

H 5.266807 0.992136 1.794424

H 6.782547 0.526732 0.982136

H 6.330866 2.259423 1.101938

**TS2**  E= -7.89975178

Nimag= -177.927

H 4.974582 -3.213245 -1.529667

S 0.483645 -0.946955 1.086459

C 1.756313 -0.887578 -0.112427

H 2.420177 -5.868614 0.591856

S 1.727289 -2.077903 -1.265086

S 2.979502 0.377595 0.095436

O 4.727885 -3.909584 -2.161916

O 3.116050 -5.425188 -2.470763

H 1.422887 -4.451004 0.942562

H -0.181634 4.590419 1.290793

H 1.229312 -5.343478 -0.593636

C 1.947734 -4.979402 0.143628

C 2.990188 -4.122193 -0.487123

H 3.616635 -3.532215 0.177588

C 3.586743 -4.548013 -1.767752

C 0.599782 0.505200 2.198643

H 0.079120 0.140841 3.092214

H 1.643765 0.658394 2.484534

C -0.056148 1.786590 1.679274

H 0.432627 2.099476 0.751097

H -1.100341 1.571939 1.423316

C 0.000866 2.932015 2.698768

H -0.480937 2.612968 3.632577

H 1.048632 3.139954 2.952903

C -0.662650 4.218095 2.202051

H -1.723089 4.060397 1.976565

H -0.598660 5.008636 2.956789

C 4.143323 0.114230 -1.299906

H 3.719285 -0.738231 -1.840965

C 4.230011 1.341304 -2.206392

H 3.253083 1.535835 -2.655111

H 4.554898 2.234038 -1.669133

H 4.944587 1.150431 -3.014640

C 5.464751 -0.365583 -0.703820

O 5.780334 -1.535410 -0.597401

O 6.239184 0.659334 -0.298287

C 7.503017 0.304080 0.304851

H 7.341423 -0.291138 1.207069

H 8.116581 -0.264297 -0.398196

H 7.979357 1.253127 0.550823

**PC**  E= -7.91107536

H 4.433411 -3.039187 -2.185242

S 1.212315 0.531329 3.301731

C 2.022230 -0.212592 1.941847

H 4.788715 -7.371959 -1.406014

S 2.130989 -1.854944 1.950033

S 2.658638 0.874003 0.716698

O 4.113957 -3.324107 -3.059068

O 3.450077 -5.191660 -4.100183

H 3.134354 -7.060475 -0.917418

H -1.270374 4.555730 0.339238

H 3.511336 -7.271966 -2.648660

C 3.869203 -6.848667 -1.708934

C 4.105347 -5.393875 -1.832765

H 4.467337 -4.847719 -0.964039

C 3.860764 -4.670121 -3.073825

C 1.134274 2.349402 3.072393

H 1.070815 2.702930 4.108367

H 2.091906 2.700894 2.679030

C -0.054749 2.859208 2.255680

H -0.012649 2.442032 1.244448

H -0.981619 2.485412 2.706530

C -0.101336 4.390862 2.175513

H -0.135057 4.807873 3.190859

H 0.830575 4.759197 1.726608

C -1.291577 4.919362 1.372515

H -2.243794 4.606309 1.814700

H -1.286198 6.013641 1.338457

C 3.425481 -0.232280 -0.514401

H 2.722258 -1.051124 -0.680430

C 3.612894 0.569662 -1.815537

H 2.649316 0.941666 -2.171069

H 4.286618 1.419395 -1.675836

H 4.030803 -0.084195 -2.584960

C 4.746612 -0.893575 -0.105940

O 5.122937 -1.939860 -0.599308

O 5.459994 -0.175837 0.771311

C 6.735698 -0.730301 1.162670

H 6.594382 -1.699160 1.647482

H 7.385855 -0.848887 0.292446

H 7.156372 -0.009049 1.863148

**P**  E= -5.84403627

S 3.227876 1.400117 3.248931

C 3.735993 0.197195 2.087313

S 3.862501 -1.347561 2.637893

S 4.073228 0.787886 0.459044

H 0.176410 4.424745 -0.313867

C 3.106676 3.046851 2.448838

H 3.267295 3.718794 3.300348

H 3.958301 3.181984 1.777045

C 1.771284 3.351666 1.767190

H 1.595385 2.629661 0.963510

H 0.961621 3.211577 2.493185

C 1.712295 4.775107 1.197108

H 1.898780 5.498290 2.002270

H 2.526388 4.909526 0.472841

C 0.378429 5.102454 0.522916

H -0.457291 5.019380 1.226543

H 0.379051 6.123845 0.128608

C 4.594779 -0.711739 -0.461792

C 3.505884 -1.214115 -1.404858

H 2.604090 -1.457760 -0.839554

H 3.263491 -0.474626 -2.170218

H 3.850864 -2.123000 -1.910088

C 5.891455 -0.352838 -1.190597

O 5.954709 0.100107 -2.311849

O 6.962016 -0.618853 -0.406461

C 8.248658 -0.282257 -0.963703

H 8.427104 -0.840123 -1.886672

H 8.307608 0.788916 -1.174770

H 8.974571 -0.562491 -0.200138

H 4.807560 -1.443214 0.325028

**carboxyl rad** E = -2.06129925

H 3.260692 -2.096917 2.028945

H 5.368238 -3.354771 5.680439

O 2.525442 -2.629188 2.363349

O 1.945718 -3.664063 4.261892

H 4.550559 -1.904554 6.229640

H 3.644892 -3.439393 6.138258

C 4.403268 -2.827154 5.648178

C 4.018128 -2.516513 4.255331

H 4.696047 -1.913263 3.652084

C 2.769676 -2.986992 3.672043

**DEPROTONATED**

**BTPA**  E= -5.20202963

S 0.004660 -1.714195 1.505970

C 1.425475 -1.995691 0.441616

H 3.526742 -5.002905 -2.071075

S 0.795835 -2.648499 -1.037191

S 2.987128 -1.634410 0.858955

O 0.557354 -3.046031 -3.693644

O 2.614507 -3.846275 -4.261407

H 3.130511 -4.542862 -0.396257

H 1.404511 3.592711 2.291514

H 1.936066 -5.400091 -1.400952

C 2.731277 -4.647055 -1.409443

C 2.224223 -3.321365 -1.970420

H 3.011185 -2.564188 -1.908236

C 1.739464 -3.416220 -3.480344

C 0.594576 -0.557439 2.776465

H 0.000236 -0.803596 3.664442

H 1.638850 -0.822258 2.974356

C 0.458490 0.922761 2.413364

H 1.035633 1.114858 1.502652

H -0.588709 1.144565 2.172529

C 0.939340 1.858100 3.530072

H 0.370519 1.654532 4.448347

H 1.987095 1.629341 3.764732

C 0.815037 3.343157 3.180949

H -0.225355 3.620385 2.974353

H 1.170026 3.976286 4.002409

**RC**  E= -7.84988631

S 3.801983 0.063779 -4.252062

C 2.500829 -0.803496 -3.411897

H 1.738590 -3.481886 -1.752494

S 2.353870 -0.503788 -1.712923

S 1.575916 -1.756756 -4.406608

O -0.694331 -1.317803 0.570815

O 1.093039 0.088777 0.673038

H 2.178738 -3.025814 -0.087996

H 5.009693 5.276073 -0.840687

H 0.517857 -3.523584 -0.458908

C 1.388177 -2.983988 -0.844526

C 0.963159 -1.540533 -1.113457

H 0.205330 -1.514516 -1.900625

C 0.392722 -0.835768 0.185164

C 4.721830 1.108362 -3.056385

H 4.856316 0.542098 -2.130923

H 5.708601 1.198337 -3.527872

C 4.124342 2.491519 -2.789136

H 4.022604 3.026661 -3.741749

H 3.114657 2.375294 -2.384492

C 4.966030 3.325735 -1.815045

H 5.058791 2.782684 -0.865849

H 5.986846 3.432385 -2.209010

C 4.382020 4.713568 -1.541152

H 4.300862 5.303543 -2.461874

H 3.380430 4.641340 -1.103708

C -1.581939 0.018908 3.830992

H -1.739984 -0.800251 3.135807

C -0.958281 1.251587 3.318885

H -1.659659 1.783250 2.653307

H -0.662324 1.931290 4.120976

H -0.111864 0.996555 2.663842

C -1.982043 -0.152360 5.203501

O -1.879073 0.664034 6.116983

O -2.534834 -1.412239 5.404336

C -2.959541 -1.682442 6.737990

H -3.358301 -2.699459 6.721411

H -2.124180 -1.618392 7.444573

H -3.736423 -0.979898 7.061230

**TS1**  E= -7.83390928

Nimag= -143.339

S -0.157375 0.402642 -2.297612

C -0.144118 -0.647653 -0.867325

H 0.203444 -3.720136 0.283795

S 1.386212 -0.914854 -0.095201

S -1.668382 -1.162040 -0.403173

O 2.126583 -2.287732 3.458113

O 3.070102 -0.819124 2.001597

H 1.970800 -3.803258 0.475561

H 4.647594 4.428040 -1.323317

H 0.919086 -4.069519 1.876325

C 1.036591 -3.491715 0.954564

C 1.079290 -2.013497 1.334795

H 0.116424 -1.707914 1.751974

C 2.219636 -1.666616 2.379538

C 1.519235 1.099004 -2.542288

H 2.252654 0.297480 -2.420004

H 1.510207 1.369302 -3.605758

C 1.865912 2.314416 -1.678843

H 1.122409 3.101717 -1.856039

H 1.789397 2.039772 -0.622916

C 3.273182 2.858168 -1.956836

H 4.006285 2.061382 -1.777602

H 3.362205 3.125292 -3.019698

C 3.636200 4.071303 -1.097283

H 2.944291 4.904935 -1.266764

H 3.602613 3.822947 -0.031132

C -2.966595 0.665107 0.466836

H -2.253400 1.416558 0.143169

C -4.257569 0.576996 -0.287746

H -4.083752 0.624064 -1.365672

H -4.793760 -0.347583 -0.055904

H -4.928951 1.407616 -0.016311

C -2.981780 0.395567 1.898220

O -3.867492 -0.186819 2.517231

O -1.837134 0.867817 2.503761

C -1.684200 0.541157 3.888429

H -0.694004 0.904806 4.168039

H -2.455698 1.027008 4.496929

H -1.739250 -0.539453 4.048557

**I**  E= -7.84261750

S -0.177321 0.199794 -2.561018

C -0.443818 -0.910122 -1.227308

H 0.723702 -4.197854 1.194169

S 0.749294 -2.115098 -0.889044

S -2.066639 -1.101236 -0.635652

O 2.112964 -0.680911 2.565606

O 3.060812 -0.633524 0.499831

H 2.417659 -3.797918 0.837606

H 3.584599 4.521160 0.144835

H 1.749018 -3.439139 2.436806

C 1.518608 -3.463915 1.367569

C 1.108521 -2.054216 0.928765

H 0.168026 -1.782051 1.409151

C 2.222301 -0.992399 1.351633

C 1.413167 1.023529 -2.166808

H 2.065313 0.266261 -1.723920

H 1.817773 1.295305 -3.149579

C 1.305071 2.246648 -1.256181

H 0.639038 2.992077 -1.712503

H 0.845820 1.942200 -0.310702

C 2.672298 2.878063 -0.960330

H 3.311869 2.118944 -0.497523

H 3.154728 3.169002 -1.904943

C 2.589976 4.098531 -0.040392

H 1.966963 4.892530 -0.471783

H 2.161679 3.829414 0.931411

C -2.620880 0.576574 0.013774

H -1.881948 1.273225 -0.382686

C -4.016765 0.898961 -0.504993

H -4.012620 0.924047 -1.597171

H -4.747058 0.157740 -0.171389

H -4.344381 1.876226 -0.130641

C -2.547345 0.553831 1.528390

O -3.466319 0.259690 2.272654

O -1.302778 0.889353 1.940139

C -1.020483 0.783291 3.349461

H 0.065051 0.728343 3.421685

H -1.416517 1.659943 3.874708

H -1.475510 -0.117658 3.766319

**TS2**  E= -7.81986124

Nimag= -71.34

S -0.412016 0.983757 -1.799233

C -0.849906 -0.621407 -1.211372

H 2.215927 -3.811259 0.458520

S 0.179103 -1.878729 -0.925243

S -2.601758 -0.847278 -1.044378

O 1.439567 -0.718198 3.381991

O 2.483852 0.093178 1.530940

H 3.380954 -2.465878 0.606921

H 4.953166 4.149075 -1.192444

H 3.035958 -3.510232 1.992626

C 2.581668 -3.013443 1.115980

C 1.495949 -2.089680 1.548670

H 0.547714 -2.531969 1.840596

C 1.835373 -0.764083 2.177022

C 1.390941 1.082581 -1.545984

H 1.621154 0.614968 -0.585071

H 1.874774 0.503199 -2.338234

C 1.853083 2.540381 -1.564497

H 1.568556 3.024057 -2.509883

H 1.340074 3.087062 -0.763930

C 3.369542 2.661240 -1.362640

H 3.639340 2.161461 -0.426079

H 3.886564 2.116546 -2.164635

C 3.867114 4.108061 -1.335570

H 3.635883 4.635167 -2.269826

H 3.406608 4.671139 -0.515431

C -3.286177 0.615120 -0.093853

H -2.525115 1.391764 -0.174930

C -4.607161 1.068768 -0.702620

H -4.454761 1.382053 -1.738250

H -5.352913 0.270271 -0.681220

H -5.014210 1.913947 -0.135161

C -3.415529 0.198099 1.364937

O -4.428933 -0.230270 1.886268

O -2.233737 0.360813 1.993563

C -2.133936 -0.115304 3.351090

H -1.071958 -0.079429 3.591466

H -2.718559 0.526231 4.019274

H -2.508805 -1.139607 3.422219

**carboxyl rad** E = -1.96328677

H 5.295737 -3.418374 5.794150

O 2.508026 -2.468908 2.468329

O 2.085156 -3.879607 4.214428

H 4.605360 -1.846092 6.183164

H 3.557913 -3.299724 6.145398

C 4.393712 -2.793386 5.652729

C 4.061852 -2.585596 4.215542

H 4.786610 -2.072300 3.580351

C 2.774862 -3.020085 3.582451

# References

1. Lorandi F., Fantin M., Isse A.A., Gennaro A. and Matyjaszewski K., Macromolecules 2019, 52, 1479−1488
2. Amatore, C.; Capobianco, G.; Farnia, G.; Sandona, G.; Saveant, J. M.; Severin, M. G.; Vianello, J. Am.Chem. Soc. 1985, 107, 1815−1824.
3. Velde, G.; Bickelhaupt, F. M.; Baerends, E. J.; Fonseca Guerra, C.; van Gisbergen, S. J. A.; Snijders, J. G.; Ziegler, T. Chemistry with ADF. *J Comput Chem* **2001**, *22* (9), 931–967. https://doi.org/10.1002/jcc.1056.
4. *ADF2019, AMS2020, SCM. Theoretical Chemistry, Vrije Universiteit, Amsterdam: The Netherlands.* https://www.scm.com/ (accessed 2024-08-30).
5. Van Lenthe, E.; Baerends, E. J.; Snijders, J. G. Relativistic Total Energy Using Regular Approximations. *J Chem Phys* **1994**, *101* (11), 9783–9792. https://doi.org/10.1063/1.467943.
6. Handy, N. C.; Cohen, A. J. Left-Right Correlation Energy. *Mol Phys* **2001**, *99* (5), 403–412. https://doi.org/10.1080/00268970010018431.
7. Lee, C.; Yang, W.; Parr, R. G. Development of the Colle-Salvetti Correlation-Energy Formula into a Functional of the Electron Density. *Phys Rev B* **1988**, *37* (2), 785–789. https://doi.org/10.1103/PhysRevB.37.785.
8. Zeppilli, D.; Madabeni, A.; Sancineto, L.; Bagnoli, L.; Santi, C.; Orian, L. Role of Group 12 Metals in the Reduction of H_2_O_2_ by Santi’s Reagent: A Computational Mechanistic Investigation. *Inorg Chem* **2023**, *62* (42), 17288–17298. https://doi.org/10.1021/acs.inorgchem.3c02568.
9. Madabeni, A.; Dalla Tiezza, M.; Omage, F. B.; Nogara, P. A.; Bortoli, M.; Rocha, J. B. T.; Orian, L. Chalcogen–Mercury Bond Formation and Disruption in Model Rabenstein’s Reactions: A Computational Analysis. *J Comput Chem* **2020**, *41* (23), 2045–2054. https://doi.org/10.1002/jcc.26371.
10. Bortoli, M.; Wolters, L. P.; Orian, L.; Bickelhaupt, F. M. Addition–Elimination or Nucleophilic Substitution? Understanding the Energy Profiles for the Reaction of Chalcogenolates with Dichalcogenides. *J Chem Theory Comput* **2016**, *12* (6), 2752–2761. https://doi.org/10.1021/acs.jctc.6b00253.
11. Bortoli, M.; Zaccaria, F.; Dalla Tiezza, M.; Bruschi, M.; Fonseca Guerra, C.; Bickelhaupt, F. M.; Orian, L. Oxidation of Organic Diselenides and Ditellurides by H_2_O_2_ for Bioinspired Catalyst Design. *Physical Chemistry Chemical Physics* **2018**, *20* (32), 20874–20885. https://doi.org/10.1039/C8CP02748J.
12. Bortoli, M.; Bruschi, M.; Swart, M.; Orian, L. Sequential Oxidations of Phenylchalcogenides by H _2_ O _2_ : Insights into the Redox Behavior of Selenium *via* DFT Analysis. *New Journal of Chemistry* **2020**, *44* (17), 6724–6731. https://doi.org/10.1039/C9NJ06449D.
13. Zhao, Y.; Truhlar, D. G. The M06 Suite of Density Functionals for Main Group Thermochemistry, Thermochemical Kinetics, Noncovalent Interactions, Excited States, and Transition Elements: Two New Functionals and Systematic Testing of Four M06-Class Functionals and 12 Other Functionals. *Theor Chem Acc* **2008**, *120* (1–3), 215–241. https://doi.org/10.1007/s00214-007-0310-x.
14. Klamt, A. Conductor-like Screening Model for Real Solvents: A New Approach to the Quantitative Calculation of Solvation Phenomena. *J Phys Chem* **1995**, *99* (7), 2224–2235. https://doi.org/10.1021/j100007a062.
15. Becke, A. D. Density-Functional Exchange-Energy Approximation with Correct Asymptotic Behavior. *Phys Rev A  (Coll Park)* **1988**, *38* (6), 3098–3100. https://doi.org/10.1103/PhysRevA.38.3098.
16. Perdew, J. P. Density-Functional Approximation for the Correlation Energy of the Inhomogeneous Electron Gas. *Phys Rev B* **1986**, *33* (12), 8822–8824. https://doi.org/10.1103/PhysRevB.33.8822.
17. Agostini, A.; Dal Farra, M. G.; Paulsen, H.; Polimeno, A.; Orian, L.; Di Valentin, M.; Carbonera, D. Similarity and Specificity of Chlorophyll *b* Triplet State in Comparison to Chlorophyll *a* as Revealed by EPR/ENDOR and DFT Calculations. *J Phys Chem B* **2019**, *123* (39), 8232–8239. https://doi.org/10.1021/acs.jpcb.9b07912.
18. Becke, A. D. A New Mixing of Hartree–Fock and Local Density‐functional Theories. *J Chem Phys* **1993**, *98* (2), 1372–1377. https://doi.org/10.1063/1.464304.
19. Grimme, S. Density Functional Theory with London Dispersion Corrections. *WIREs Computational Molecular Science* **2011**, *1* (2), 211–228. https://doi.org/10.1002/wcms.30.
20. Grimme, S.; Ehrlich, S.; Goerigk, L. Effect of the Damping Function in Dispersion Corrected Density Functional Theory. *J Comput Chem* **2011**, *32* (7), 1456–1465. https://doi.org/10.1002/jcc.21759.
21. Becke, A. D.; Johnson, E. R. A Density-Functional Model of the Dispersion Interaction. *J Chem Phys* **2005**, *123* (15), 154101. https://doi.org/10.1063/1.2065267.
22. Rehm, D.; Weller, A. Kinetics of Fluorescence Quenching by Electron and H-Atom Transfer. *Israel Journal of Chemistry* **1970**, *8*, 259.
23. Marcus, R. A. On the theory of oxidation‐reduction reactions involving electron transfer. I. *The Journal of chemical physics* **1956**, *24*, 966.
24. Valencia, D. P.; González, F. J. Estimation of diffusion coefficients by using a linear correlation between the diffusion coefficient and molecular weight. *Journal of Electroanalytical Chemistry* **2012**, *681*, 121.
